# Analysis of the common genetic component of large-vessel vasculitides through a meta-Immunochip strategy

**DOI:** 10.1038/srep43953

**Published:** 2017-03-09

**Authors:** F. David Carmona, Patrick Coit, Güher Saruhan-Direskeneli, José Hernández-Rodríguez, María C. Cid, Roser Solans, Santos Castañeda, Augusto Vaglio, Haner Direskeneli, Peter A. Merkel, Luigi Boiardi, Carlo Salvarani, Miguel A. González-Gay, Javier Martín, Amr H. Sawalha, Spanish GCA Study Group, Agustín Martínez-Berriochoa, Ainhoa Unzurrunzaga, Ana Hidalgo-Conde, Ana Belén Madroñero Vuelta, Antonio Fernández-Nebro, M. Carmen Ordóñez-Cañizares, Benjamín Fernández-Gutiérrez, Luis Rodríguez-Rodríguez, Begoña Escalante, Begoña Marí-Alfonso, Bernardo Sopeña, Carmen Gómez-Vaquero, Enrique Raya, Elena Grau, José A. Román, Esther F. Vicente, Eugenio de Miguel, Francisco J. López-Longo, Lina Martínez, Inmaculada C. Morado, J. Bernardino Díaz-López, Luis Caminal-Montero, Aleida Martínez-Zapico, Javier Narváez, Jordi Monfort, Laura Tío, José A. Miranda-Filloy, Julio Sánchez-Martín, Juan J. Alegre-Sancho, Luis Sáez-Comet, Mercedes Pérez-Conesa, Marc Corbera-Bellalta, Marc Ramentol-Sintas, María Jesús García-Villanueva, Mercedes Guijarro Rojas, Norberto Ortego-Centeno, Raquel Ríos Fernández, José Luis Callejas, Olga Sanchez Pernaute, Patricia Fanlo Mateo, Ricardo Blanco, Sergio Prieto-González, Víctor Manuel Martínez-Taboada, Italian GCA Study Group, Alessandra Soriano, Claudio Lunardi, Davide Gianfreda, Daniele Santilli, Francesco Bonatti, Francesco Muratore, Giulia Pazzola, Olga Addimanda, Giacomo Emmi, Giuseppe A. Ramirez, Lorenzo Beretta, Marcello Govoni, Marco A. Cimmino, Turkish Takayasu Study Group, Ahmet Mesut Onat, Ayse Cefle, Ayten Yazici, Bünyamin Kısacık, Ediz Dalkilic, Emire Seyahi, Izzet Fresko, Ercan Tunc, Eren Erken, Hüseyin TE Ozer, Kenan Aksu, Gokhan Keser, Mehmet A. Ozturk, Muge Bıcakcıgil, Nurşen Duzgun, Omer Karadag, Sedat Kiraz, Ömer N. Pamuk, Servet Akar, Fatos Onen, Nurullah Akkoc, Sevil Kamali, Murat Inanc, Sibel P. Yentür, Sibel Z. Aydin, Fatma Alibaz-Oner, Timuçin Kaşifoğlu, Veli Cobankara, Zeynep Ozbalkan, Askin Ates, Yasar Karaaslan, Vasculitis Clinical Research Consortium, Simon Carette, Sharon A. Chung, David Cuthbertson, Lindsay J. Forbess, Gary S. Hoffman, Nader A. Khalidi, Curry L. Koening, Carol A. Langford, Carol A. McAlear, Kathleen McKinnon-Maksimowicz, Paul A. Monach, Larry Moreland, Christian Pagnoux, Philip Seo, Robert Spiera, Antoine G. Sreih, Kenneth J. Warrington, Steven R. Ytterberg

**Affiliations:** 1 Instituto de Parasitología y Biomedicina ‘López-Neyra’, IPBLN-CSIC, PTS Granada, Granada, Spain; 2 Departamento de Genética e Instituto de Biotecnología, Universidad de Granada, Granada 18016, Spain; 3 Division of Rheumatology, Department of Internal Medicine, University of Michigan, Ann Arbor, Michigan, USA; 4 Department of Physiology, Istanbul Medical Faculty, Istanbul University, Istanbul, Turkey; 5 Vasculitis Research Unit, Department of Autoimmune Diseases, Hospital Clínic, University of Barcelona, Institut d’Investigacions Biomèdiques August Pi i Sunyer (IDIBAPS), Barcelona, Spain; 6 Autoimmune Systemic Diseases Unit, Department of Internal Medicine, Hospital Vall d’Hebron, Autonomous University of Barcelona, Barcelona, Spain; 7 Department of Rheumatology, Hospital de la Princesa, IIS-IP, Madrid, Spain; 8 Unit of Nephrology, University Hospital of Parma, Parma, Italy; 9 Marmara University, School of Medicine, Department of Rheumatology, Istanbul, Turkey; 10 Division of Rheumatology and Department of Epidemiology and Biostatistics, University of Pennsylvania, Philadelphia, USA; 11 Rheumatology Unit, Department of Internal Medicine, Azienda Ospedaliera ASMN, Istituto di Ricovero e Cura a Carattere Scientifico, Reggio Emilia, Italy; 12 Department of Rheumatology, Hospital Universitario Marqués de Valdecilla, IDIVAL, University of Cantabria, Santander, Spain; 13 Division of Rheumatology, Department of Internal Medicine, University of Michigan, Ann Arbor, Michigan, USA Center for Computational Medicine and Bioinformatics, University of Michigan, Ann Arbor, Michigan, USA; 14 Department of Internal Medicine, Hospital de Cruces, Barakaldo, Spain; 15 Department of Internal Medicine, Hospital de Galdakano, Vizcaya, Spain; 16 Department of Internal Medicine, Hospital Universitario Virgen de la Victoria, Málaga, Spain; 17 Departament of Internal Medicine, Hospital Universitario Arnau de Vilanova, Lleida, Spain; 18 Rheumatology Department, Hospital Carlos Haya, Málaga, Spain; 19 Department of Rheumatology, Hospital Clínico San Carlos, Madrid, Spain; 20 Department of Internal Medicine, Hospital Clínico Universitario Lozano Blesa, Zaragoza, Spain; 21 Department of Internal Medicine, Corporació Sanitaria Parc Taulí, Instituto Universitario Parc Taulí, UAB, Sabadell, Barcelona, Spain; 22 Department of Internal Medicine, Complejo Hospitalario Universitario de Vigo Xeral-Chuvi, Spain; 23 Department of Rheumatology, Hospital Universitario de Bellvitge-IDIBELL, L’Hospitalet de Llobregat, Barcelona, Spain; 24 Department of Rheumatology, Hospital Clínico Universitario San Cecilio, Granada, Spain; 25 Department of Rheumatology, Hospital Universitario y Politécnico La Fe, Valencia, Spain; 26 Deptartment of Rheumatology, Hospital de La Princesa, IISIP, Madrid, Spain; 27 Department of Rheumatology, Hospital Universitario de La Paz, Madrid, Spain; 28 Department of Rheumatology, Hospital General Universitario Gregorio Marañón, Madrid, Spain; 29 Department of Rheumatology, Hospital Clínico San Carlos, Madrid, Spain; 30 Department of Internal Medicine, Hospital Central de Asturias, Oviedo, Spain; 31 Department of Rheumatology, Hospital Universitario de Bellvitge-IDIBELL, L’Hospitalet de Llobregat, Barcelona, Spain; 32 Department of Rheumatology, Grup de recerca cel• lular en inflamació i cartílag. IMIM (Institut de Recerca Hospital del Mar), Barcelona, Spain; 33 Department of Rheumatology, Hospital Xeral-Calde, Lugo, Spain; 34 Department of Rheumatology, Hospital Universitario 12 de Octubre, Madrid, Spain; 35 Departament of Reumatology, Hospital Universitario Doctor Peset, Valencia, Spain; 36 Department of Internal Medicine, Hospital Universitario Miguel Servet, Zaragoza, Spain; 37 Vasculitis Research Unit, Department of Autoimmune and Systemic Diseases, Hospital Clinic, University of Barcelona, Centre de Recerca Biomèdica Cellex (IDIBAPS), Barcelona, Spain; 38 Department of Rheumatology, Hospital Ramón y Cajal, Madrid, Spain; 39 Department de Pathology, Hospital de La Princesa, IISIP, Madrid, Spain; 40 Department of Internal Medicine, Hospital Clínico San Cecilio, Granada, Spain; 41 Rheumatology Division, Fundación Jiménez Díaz, Universidad Autónoma, Madrid, Spain; 42 Department of Internal Medicine, Hospital Virgen del Camino, Pamplona, Spain. Rheumatology Department, Hospital Universitario Marqués de Valdecilla, Facultad de Medicina, Universidad de Cantabria, Santander, Spain; 43 Department of Clinical Medicine and Rheumatology, Campus Bio-Medico University, Rome, Italy; 44 Department of Medicine, Universita degli Studi di Verona, Verona, Italy; 45 Department of Clinical and Experimental Medicine, University of Parma, School of Medicine, Parma, Italy; 46 Unit of Internal Medicine and Rheumatology, University Hospital of Parma, Parma, Italy; 47 Department of Clinical and Experimental Medicine, Medical Genetics Unit, University of Parma, Parma, Italy; 48 Department of Experimental and Clinical Medicine, University of Florence, Florence, Italy; 49 Unit of Internal Medicine and Immunology, IRCCS Ospedale San Raffaele and Università Vita-Salute San Raffaele, Milan, Italy; 50 Referral Center for Systemic Autoimmune Diseases, Fondazione IRCCS Ca’ Granda Ospedale Maggiore Policlinico di Milano, Milan, Italy; 51 Department of Rheumatology, Azienda Ospedaliero Universitaria S. Anna, University of Ferrara, Ferrara, Italy; 52 Research Laboratory and Academic Division of Clinical Rheumatology, Department of Internal Medicine, University of Genova, Genova, Italy; 53 Department of Rheumatology, Gaziantep University, Faculty of Medicine, Gaziantep, Turkey; 54 Department of Rheumatology, Kocaeli University, Faculty of Medicine, Kocaeli, Turkey; 55 Department of Rheumatology, Kocaeli University, Faculty of Medicine, Kocaeli, Turkey; 56 Department of Rheumatology, Gaziantep University, Faculty of Medicine, Gaziantep, Turkey; 57 Department of Rheumatology, Uludag University, Faculty of Medicine, Bursa, Turkey; 58 Department of Rheumatology, Istanbul University, Cerrahpasa Faculty of Medicine, Istanbul, Turkey; 59 Department of Rheumatology, Suleyman Demirel University, Faculty of Medicine, Isparta, Turkey; 60 Department of Rheumatology, Cukurova University, Faculty of Medicine, Adana, Turkey; 61 Department of Rheumatology, Cukurova University, Faculty of Medicine, Adana, Turkey; 62 Department of Rheumatology, Ege University, Faculty of Medicine, Izmir, Turkey; 63 Department of Rheumatology, Gazi University, Faculty of Medicine, Ankara, Turkey; 64 Department of Rheumatology, Yeditepe University, Faculty of Medicine, Istanbul, Turkey; 65 Department of Rheumatology, Ankara University, Faculty of Medicine, Ankara, Turkey; 66 Department of Rheumatology, Hacettepe University, Faculty of Medicine, Ankara, Turkey; 67 Department of Rheumatology, Trakya University, Faculty of Medicine, Edirne, Turkey; 68 Department of Rheumatology, Dokuz Eylül University, Faculty of Medicine, Izmir, Turkey; 69 Department of Rheumatology, Istanbul University, Istanbul Faculty of Medicine, Istanbul, Turkey; 70 Department of Physiology, Istanbul University, Istanbul Faculty of Medicine, Istanbul, Turkey; 71 Department of Rheumatology, Osman Gazi University, Faculty of Medicine, Eskişehir, Turkey; 72 Department of Rheumatology, Pamukkale University Faculty of Medicine, Denizli, Turkey; 73 Department of Rheumatology, Ankara Numune Training and Research Hospital, Ankara, Turkey; 74 Division of Rheumatology, Mount Sinai Hospital, Toronto, ON, USA; 75 Rosalind Russell-Ephraim P. Engleman Rheumatology Research Center, Division of Rheumatology, University of California San Francisco, San Francisco, CA, USA; 76 Department of Biostatistics, University of South Florida, Tampa, FL, USA; 77 Division of Rheumatology, Cedars-Sinai Medical Center, Los Angeles, CA, USA; 78 Center for Vasculitis Care and Research, Department of Rheumatology, Cleveland Clinic Foundation, Cleveland, OH, USA; 79 Division of Rheumatology, St. Joseph’s Healthcare, McMaster University, Hamilton, ON, Canada; 80 Division of Rheumatology, University of Utah, Salt Lake City, UT, USA; 81 Penn Vasculitis Center, Division of Rheumatology, University of Pennsylvania, Philadelphia, PA, USA; 82 Division of Rheumatology, University of Pittsburgh, Pittsburgh, PA, USA; 83 The Vasculitis Center Section of Rheumatology, Boston University School of Medicine, Boston, MA, USA; 84 Division of Rheumatology and Clinical Immunology, University of Pittsburgh, Pittsburgh, PA, USA; 85 Division of Rheumatology, Johns Hopkins University, Baltimore, MD, USA; 86 Department of Rheumatology, Hospital for Special Surgery, New York, USA; 87 Division of Rheumatology, Mayo Clinic College of Medicine, Rochester, MN, USA

## Abstract

Giant cell arteritis (GCA) and Takayasu’s arteritis (TAK) are major forms of large-vessel vasculitis (LVV) that share clinical features. To evaluate their genetic similarities, we analysed Immunochip genotyping data from 1,434 LVV patients and 3,814 unaffected controls. Genetic pleiotropy was also estimated. The HLA region harboured the main disease-specific associations. GCA was mostly associated with class II genes (*HLA-DRB1*/*HLA-DQA1*) whereas TAK was mostly associated with class I genes (HLA-B/MICA). Both the statistical significance and effect size of the HLA signals were considerably reduced in the cross-disease meta-analysis in comparison with the analysis of GCA and TAK separately. Consequently, no significant genetic correlation between these two diseases was observed when HLA variants were tested. Outside the HLA region, only one polymorphism located nearby the *IL12B* gene surpassed the study-wide significance threshold in the meta-analysis of the discovery datasets (rs755374, P = 7.54E-07; OR_GCA_ = 1.19, OR_TAK_ = 1.50). This marker was confirmed as novel GCA risk factor using four additional cohorts (P_GCA_ = 5.52E-04, OR_GCA_ = 1.16). Taken together, our results provide evidence of strong genetic differences between GCA and TAK in the HLA. Outside this region, common susceptibility factors were suggested, especially within the *IL12B locus*.

Vasculitides represent a heterogeneous group of complex disorders characterised by chronic inflammatory lesions of the blood vessels. Although the pathogenesis of vasculitides is far from being completely understood, cumulating data clearly suggest that both the innate and adaptive responses contribute to their development and progression^1^. Vasculitides show a large spectrum of clinical manifestations that depend on the affected blood vessel. In this regard, the Chapel Hill Consensus Conference proposed a nomenclature system in which the vasculitides were subdivided into three main groups: small-vessel, medium-vessel, and large-vessel vasculitis (LVV). The LVV group includes giant cell arteritis (GCA) and Takayasu’s arteritis (TAK), which mainly involve arteries of large calibre such as the aorta and its major branches^2^. These two forms of vasculitis develop predominantly in women, with GCA generally affecting people over 50 years of age in Western countries, especially those of European origin, and TAK affecting younger patients with a higher prevalence in Turkey, Japan, India, and China^3^,^4^.

In the last years, the use of novel technologies has produced a substantial advance in the elucidation of the genetic component of LVV^5^. Large-scale genetic analyses have been recently published separately for both GCA and TAK using the Immunochip platform^6^,^7^. The Immunochip has been shown to be one of the most successful platforms to identify immune-related risk variants for a large spectrum of immune-mediated diseases. The use of the same platform in these studies has facilitated the identification of shared aetiopathogenic pathways amongst these disorders, supporting the hypothesis of a common genetic background underlying autoimmunity^8^.

To contribute to the development of better diagnostic and prognostic markers of LVV, we evaluated the genetic similarities between GCA and TAK by performing an inter-disease meta-analysis of genomic data.

## Results

### Analysis of the HLA region

**Figure 1:**
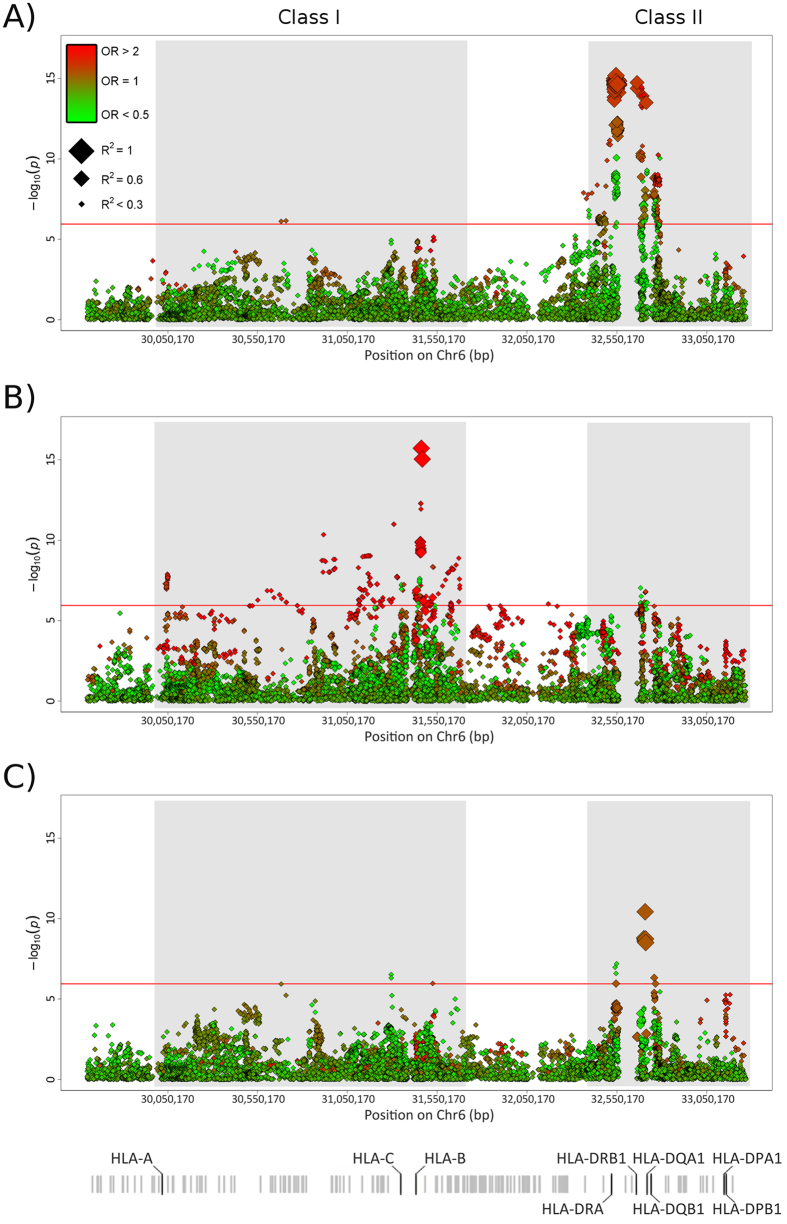
Manhattan plot representation of the results of the HLA region in (**A**) giant cell arteritis, (**B**) Takayasu’s arteritis, and (**C**) the meta-analysis of both forms of vasculitis. The log_10_ of the P values are plotted against their physical chromosomal position. A red/green color gradient was used to represent the effect size of each analysed polymorphism (red for risk and green for protection). The red line represents the study-wide level of significance (P < 1.13E-06). HLA class I and II subregions are highlighted in grey.

The HLA region harboured the main disease-specific associations in our study cohort (Fig. 1). In this context, GCA was mostly associated with class II genes, with the SNP rs9405038 (located between *HLA-DRA* and *HLA-DRB1*) representing the lead signal (P = 6.65E-16, OR = 1.60). In contrast, the main associations with TAK were located within the class I subregion, with rs12524487 (located between HLA-B and MHC class I polypeptide-related sequence A; *MICA*) as the strongest hit (P = 1.92E-16, OR = 3.70). Neither SNP showed even suggestive P-values in the analysis of the other type of vasculitis (TAK: rs9405038, P = 0.010; GCA: rs12524487, P = 0.244). As a consequence, a high heterogeneity (Q < 0.05) was observed across the region. Consequently, a random effects model was used to meta-analyse the HLA data. Although some class I and II markers surpassed the study-wide significance threshold (*e.g*. class I: rs9263969, P = 3.01E-07, OR_GCA_ = 0.77, OR_TAK_ = 0.77; class II: rs9272105, P = 3.74E-11, OR_GCA_ = 1.38, OR_TAK_ = 1.57), both the number of associations and their effect size was considerably reduced in comparison with the analysis of GCA and TAK separately (Fig. 1, see Supplementary Table S1).

### Analysis of the non-HLA region

Outside the HLA region, only one variant surpassed the study-wide significance threshold in the overall meta-analysis including both diseases (rs755374, P = 7.54E-07; OR_GCA_ = 1.19, OR_TAK_ = 1.50; Table 1, see Supplementary Figure S1). This SNP is located in an intergenic region at 71 kb 5′ of the interleukin 12B (*IL12B*) gene (see Supplementary Figure S2).

Other suggestive common susceptibility factors for both diseases that showed trends of association included glutamate ionotropic receptor NMDA type subunit 2 A (*GRIN2A*; rs1448258, P = 2.69E-06, OR_GCA_ = 1.23, OR_TAK_ = 1.29), G-protein signaling modulator 1 (*GPSM1*; rs28489139, P = 1.38E-05, OR_GCA_ = 1.27, OR_TAK_ = 1.98), nitric oxide synthase 2 (*NOS2*; rs7406657, P = 2.65E-05, OR_GCA_ = 0.76, OR_TAK_ = 0.88), ASH1 like histone lysine methyltransferase (*ASH1L*; rs7340058, P = 6.26E-05, OR_GCA_ = 0.61, OR_TAK_ = 0.58), REL proto-oncogene, NF-kB subunit (*REL*; rs79657074, P = 6.73E-05, OR_GCA_ = 1.32, OR_TAK_ = 1.82), SMG6, nonsense mediated mRNA decay factor (*SMG6*, rs10852932; P = 6.88E-05, OR_GCA_ = 0.83, OR_TAK_ = 0.80), protein kinase C theta (*PRKCQ*, rs587198; P = 7.87E-05, OR_GCA_ = 1.20, OR_TAK_ = 1.22), endoplasmic reticulum aminopeptidase 1 (*ERAP1*, rs2255637; P = 8.77E-05, OR_GCA_ = 1.18, OR_TAK_ = 1.27), and ubiquitin conjugating enzyme E2 E3 (*UBE2E3*, rs7349232; P = 9.84E-05, OR_GCA_ = 1.24, OR_TAK_ = 1.25).

As previously described^7^, a group of variants in high linkage disequilibrium (LD), located downstream of the proteasome assembly chaperone 1 (*PSMG1*) gene on chromosome 21q22, also showed evidence of association with TAK in the analyses of each disease separately (lead variant: rs35819975, P = 7.98E-07, OR = 0.62).

### Additional analyses of the association of IL12B with large-vessel vasculitis

To further analyse the consistency of the putative shared association with the *IL12B* variant rs755374, we checked the signal in the remaining cohorts included in the published GCA Immunochip, which comprised 650 additional cases of GCA and 12,491 controls from UK, North America (USA/Canada), Germany, and Norway^6^ (see Supplementary Table S2). Significant results at the nominal level of significance were observed when these replication cohorts were tested for *IL12B* rs755374 (P = 4.69E-02, OR = 1.13, 95% CI = 1.01–1.27), as well as when a meta-analysis including all GCA cohorts was performed (P = 5.52E-04, OR = 1.16, 95% CI = 1.07–1.26). Finally, an overall P = 3.41E-07 was obtained after meta-analysing all the available data for this SNP (including the six GCA cohorts and the two TAK cohorts), with no heterogeneity observed amongst the different ORs (Q = 0.19).

To further understand this common association, we looked for SNPs in high LD (r^2^ > 0.8) with *IL12B* rs755374 in the European populations of the 1000 genomes project using the online annotation tool HaploReg v4.1 (http://www.broadinstitute.org/mammals/haploreg/haploreg.php)^9^. Three markers were identified (rs6871626, rs56167332, and rs4921492), all of them previously associated with other immune-mediated diseases (Table 2). Interestingly, different functional annotations were observed for rs4921492, including enhancer and promoter histone marks (H3K4me1 and H3K4me3, respectively) as well as DNAse hypersensitivity peaks in different immune cell types. Additionally, the associated hit of our study, rs755374, also overlapped with the H3K4me1 enhancer histone mark in primary B cells from peripheral blood. Furthermore, the “genome-wide repository of associations between SNPs and phenotypes”^10^ showed 589 expression quantitative trait *loci* (eQTL) hits for rs6871626 in normal prepouch ileum, including key genes of the immune response like *CD40, IL2RA, IL6R, IL10RA, IL12RB1*, and different HLA class II molecules.

### Genetic correlation between giant cell arteritis and Takayasu’s arteritis

We estimated the whole genetic overlap between these two forms of LVV using a bivariate REML analysis on the Immunochip data (Table 3). A significant correlation was suggested only outside the HLA region (rG = 0.500, SE = 0.194, P = 5.00E-03) but not inside the region (rG = 0.012, SE = 0.192, P = 0.5). Similar results were obtained when we quantified the correlation by analysing polygenic risk scores on one disease calculated with the ORs of the markers that showed suggestive P-values (P < 1.00E-04) on the other disease (Table 3). GCA cases had a significant enrichment of non-HLA risk alleles for TAK when compared to controls (P_GCA_ = 3.53E-03) and vice-versa (P_TAK_ = 3.60E-02), with no correlation observed within the HLA region (P_GCA_ = 0.27 and P_TAK_ = 0.70).

## Discussion

This cross-disease analysis of Immunochip data represents the first interrogation of the genetic overlap between GCA and TAK. Although both conditions are characterised by inflammatory damage of the wall of large arteries^2^, the patterns of vascular involvement differ somewhat between them. In TAK the most affected vessels correspond with the aorta and its major branches, whereas in GCA the main lesions are usually localised in more peripheral arteries (such as the branches of the external carotid artery) and GCA is sometimes associated with the development of polymyalgia rheumatica^11^. Despite the evident differences that these two types of LVV show in the clinical manifestations, geographic distributions, and average age of disease onset, their similar histopathological features (with presence of inflammatory infiltrates within the vessel walls and granulomatous lesions^12^) have raised controversy over whether or not these conditions represent different subtypes of a single disease entity^3^. Comparative analyses of their genetic components may definitively help to answer this question.

Our results support the existence of a shared portion of the genetic susceptibility between GCA and TAK, but only outside the HLA region. As previously described^6^, GCA is mostly associated with class II genes (*HLA-DRB1*/*HLA-DQA1*), although some less intense class I signals may be also involved in disease predisposition. The opposite is observed in TAK, that is, the peak HLA associations are located within class I (*HLA-B*/*MICA*), with lower but still significant signals in class II^7^,^13^. The meta-analysis of this genomic region in our study cohorts reduced considerably the statistical significance of the disease-specific associations, thus confirming that distinct HLA haplotypes define each form of LVV. In this sense, GCA can be grouped with vasculitides such as ANCA-associated vasculitis or IgA vasculitis into class II diseases associated with *HLA-DRB1* alleles^14^,^15^, while TAK and Behçet’s disease would represent archetypal class I diseases^7^,^16^.

Despite the similar histological features of GCA and TAK (which may be a consequence of the activation of dendritic cells within the vessel wall^3^,^17^), the different genetic architecture between these two diseases within the HLA region may reflect distinctive effects of the initial inflammatory stimuli. In this context, whereas the infiltrates in GCA are mostly composed of CD4+ T cells and macrophages^12^, infiltrations of CD8+ T cells are characteristic in TAK lesions^18^, which is in agreement with their specific associations with the HLA class II and I *loci*, respectively. Indeed, early studies described an increased *in vitro* cytotoxicity and a direct action of CD8+ T cells on large arteries from TAK patients^19^.

Regarding the non-HLA region, different relevant genes for the development of autoimmunity processes were suggested as shared risk factors for LVV, including *NOS2, ERAP1, REL* and *PRKQC*, which have been associated with psoriasis, Behçet disease, ankylosing spondylitis (AS), and rheumatoid arthritis, amongst others^20^,^21^,^22^. In the case of *NOS2*, which encodes a nitric oxide (NO) synthase involved in the release of NO during the immune response, previously published genetic evidences supported a role of this gene in GCA pathogenesis^23^,^24^.

However, a SNP located 5′ of *IL12B*, rs755374, represented the most consistent common associated signal between GCA and TAK. *IL12B* is a well-established risk gene for TAK^7^,^13^,^25^, but this is the first time that it has been implicated in the predisposition of GCA. Although it should be noted that this genetic variant represented a suggestive signal in the original Immunochip of this disease (P = 5.52E-04, OR = 1.16)^6^. This gene encodes the P40 subunit that is shared between the interleukins IL-12 and IL-23. It has been described that IL-12 induces Th1 differentiation, whereas IL-23 along with IL-1β promote Th-17 differentiation and function^26^. Consistent with the association with *IL12B* reported here, previous candidate gene studies have reported genetic associations between GCA and receptors of these cytokines^27^. Increasing evidence points to Th-1 and Th-17 cells as pivotal players in the development of LVV^12^,^28^. Specifically, in GCA, recent studies have shown that these cell types are directly involved in the main immunopathological pathways responsible for the clinical phenotypes of this type of vasculitis^29^,^30^,^31^,^32^,^33^,^34^. Interestingly, blocking of IL-12/23 p40 with ustekinumab resulted in an improvement of symptoms in patients with refractory GCA^35^.

The associated *IL12B* SNP is in high LD (r2 > 0.9) with other *IL12B* variants (rs6871626, rs56167332, and rs4921492) that overlap with different regulatory marks in immune cells (Table 2). One of them, rs6871626, has been recently established as a marker for disease severity in TAK^25^. These proxies have been previously identified as key susceptibility factors for several immune-mediated diseases, including TAK, inflammatory bowel diseases (both Crohn’s disease and ulcerative colitis), AS, and sarcoidosis, and leprosy^7^,^13^,^36^,^37^,^38^,^39^,^40^,^41^.

In summary, through an inter-disease meta-analysis of large scale genotyping data we evaluated the extent of genetic similarities between GCA and TAK. Our results suggest that the genetic architecture of these disorders differs more than expected, especially in the HLA region, considering their similar patterns of histological disease. Nevertheless, common non-HLA associations were suggested, including *IL12B*. Given that these conditions are often diagnosed after periods of low-level symptoms or even no symptoms, these data may lead to both reliable disease-specific diagnostic molecular markers and more targeted therapies for each form of LVV.

## Methods

### Study population

In total, 1,434 patients diagnosed with LVV and 3,814 unaffected controls were analysed. The study cohort comprised the two populations of patients with TAK included in the Immunochip analysis^7^, one of European ancestry from North America (USA/Canada; 110 TAK cases and 558 unaffected controls) and one from Turkey (327 TAK cases and 481 unaffected controls), as well as two of the six cohorts included in the Immunochip analysis of GCA^6^, a cohort from Spain (759 GCA cases and 1,505 unaffected controls) and a cohort from Italy (238 GCA cases and 1,270 unaffected controls) (see Supplementary Figure S3). The reason for not including all the available datasets of the Immunochip of GCA was to maintain a balance between the sample sizes of both diseases. All cases were diagnosed following the 1990 American College of Rheumatology classification criteria for both TAK and GCA^42^,^43^. The main clinical features of the analysed patients were detailed elsewhere^6^,^7^. All participants signed a written informed consent before being included in the study, and the procedures were followed in accordance with the ethical standards of the Ethics Committees on human experimentation of Consejo Superior de Investigaciones Científicas (Spain), University of Cantabria (Spain), Hospital Clínic de Barcelona (Spain), University of Parma (Italy), University of Michigan (USA), Marmara University (Turkey), and Istanbul University (Turkey), which provided approval for the study and all experimental protocols.

### Quality control and data imputation

To ensure consistency amongst datasets, different standard quality filters were applied to the Immunochip raw data of both diseases in parallel with PLINK v1.07^44^ prior imputation: single-nucleotide polymorphisms (SNPs) with cluster separation <0.4, call rates <98%, minor allele frequencies (MAF) <1%, and those deviating from Hardy-Weinberg equilibrium (HWE; P < 0.001) were excluded; samples with <95% successfully called SNPs, first-degree relatives (identity by descent >0.4), and those showing >4 standard deviations from the cluster centroids of each population using the first ten principal components (PC; estimated using the ancestry markers included in the Immunochip) were also removed. Sex chromosomes were not analysed.

SNP genotype imputation was performed separately for each dataset using IMPUTE v.2^45^ and the 1000 Genome Project Phase III data as reference panel (www.1000genomes.org)^46^. For that, the SNP map was updated to rs# and build 37 (HG19) using PLINK. Subsequently, chunks of 50,000 Mbp were generated and imputed with a probability threshold of 0.9 for merging genotypes. SNP data were also tightly filtered in PLINK after imputation as follows: call rate <98%, MAF <1%, HWE P < 0.001. A total of 213,188 SNPs were shared amongst the different imputed studies after QC.

### Statistical Analysis

All analyses were carried out with PLINK and the R-base software under GNU Public license v2. First, each case-control study was tested for association by logistic regression on the best-guess genotypes (>0.9 probability) assuming an additive model and using the ten first PCs and gender as covariates. Next, all studies were meta-analysed with the inverse variance weighted meta-analysis method under a fixed effects models, except for the HLA region that was analysed under a random effects model. Cochran’s Q test was used to measure the heterogeneity of the ORs amongst the different datasets. The threshold for statistical significance in our study was established at 1.13E-06, accordingly with the estimation by the genetic type I error calculator software, which implements a Bonferroni-based validated method to control for type I errors^47^.

### Analysis of the Genetic Pleiotropy

The genetic pleiotropy between GCA and TAK was assessed using both a bivariate and a polygenic risk score (PRS) analysis on Immunochip data, as previously described^48^. In brief, the genetic correlation (rG) was estimated by GCTA bivariate restricted maximum likelihood (REML) analysis using a genetic relationship matrix, containing data of identity by descent relationship for all pair-wise sets of individuals, and the first ten PCs as covariates. The statistical significance was determined by a likelihood ratio test (LRT). The genetic overlap between both types of vasculitis was also calculated by analysing PRS in one disease predicting risk for the other disease. We obtained for each participant included in the GCA/control cohorts a weighted mean of genotype dosage using the log of the ORs of set of tag SNPs (r2 < 0.20 within 500 kb windows) showing suggestive P-values in the TAK meta-analysis (P < 1.00E-04), and vice versa. We then analysed the difference between the score distribution in case and control subjects (considering the first ten PCs, country of origin, and gender as variables) through a LRT to quantify the relationship between the computed scores and disease status.

## Additional Information

**How to cite this article**: Carmona, F. D. *et al*. Analysis of the common genetic component of large-vessel vasculitides through a meta-Immunochip strategy. *Sci. Rep.* **7**, 43953; doi: 10.1038/srep43953 (2017).

**Publisher's note:** Springer Nature remains neutral with regard to jurisdictional claims in published maps and institutional affiliations.

## Supplementary Material

Supplementary Information

## Figures and Tables

**Table 1:** Suggestive shared signals (P < 1E-04) between giant cell arteritis and Takayasu’s arteritis outside the HLA region.

Chr	SNP	BP (GRCh37)	*Locus*	Change	META LVV		META GCA			META TAK		
					P	Q	OR [95% CI]	P	Q	OR [95% CI]	P	Q
5	rs755374	158,829,294	*IL12B*	T < C	7.54E-07	0.14	1.19 [1.06–1.33]	3.92E-03	0.69	1.50 [1.26–1.78]	4.71E-06	0.47
16	rs1448258	10,151,357	*GRIN2A*	T < C	2.69E-06	0.54	1.23 [1.10–1.37]	1.70E-04	0.37	1.29 [1.08–1.53]	4.48E-03	0.28
9	rs28489139	139,232,033	*GPSM1*	G < A	1.38E-05	0.10	1.27 [1.04–1.55]	1.71E-02	0.40	1.98 [1.45–2.69]	1.52E-05	0.80
17	rs7406657	26,083,690	*NOS2*	C < G	2.65E-05	0.62	0.76 [0.66–0.86]	2.80E-05	0.79	0.88 [0.73–1.06]	1.87E-01	0.80
17	rs4796017	26,074,991	*NOS2*	G < A	3.58E-05	0.26	0.79 [0.71–0.88]	2.73E-05	0.17	0.91 [0.77–1.07]	2.48E-01	0.61
17	rs7207044	26,075,524	*NOS2*	A < G	3.81E-05	0.21	0.79 [0.70–0.88]	2.56E-05	0.14	0.91 [0.77–1.08]	2.67E-01	0.56
2	rs17438590	185,948,301	*LOC105373782*	A < T	4.86E-05	0.74	0.73 [0.60–0.89]	1.38E-03	0.79	0.68 [0.51–0.92]	1.14E-02	0.31
1	rs7340058	155,334,933	*ASH1L*	A < G	6.26E-05	0.62	0.61 [0.45–0.83]	1.70E-03	0.74	0.58 [0.38–0.89]	1.28E-02	0.20
2	rs58794562	185,949,321	*LOC105373782*	T < A	6.36E-05	0.71	0.74 [0.61–0.89]	1.83E-03	0.74	0.68 [0.51–0.92]	1.10E-02	0.30
17	rs9898308	26,059,738	*NOS2*	G < T	6.50E-05	0.29	0.79 [0.71–0.89]	4.50E-05	0.21	0.91 [0.77–1.08]	2.80E-01	0.57
17	rs4796023	26,078,694	*NOS2*	C < T	6.59E-05	0.07	0.78 [0.70–0.87]	1.50E-05	0.06	0.94 [0.80–1.11]	4.79E-01	0.58
2	rs79657074	61,116,590	*REL*	T < A	6.73E-05	0.28	1.32 [1.01–1.72]	4.42E-02	0.29	1.82 [1.33–2.48]	1.62E-04	0.55
17	rs10852932	2,143,460	*SMG6*	T < G	6.88E-05	0.24	0.83 [0.74–0.93]	1.53E-03	0.64	0.80 [0.66–0.96]	1.45E-02	0.05
4	rs4032303	67,463,707	*Intergenic*	T < C	7.01E-05	0.20	1.32 [1.16–1.50]	2.54E-05	0.51	1.09 [0.89–1.32]	4.09E-01	0.21
7	rs2690884	31,307,585	*Intergenic*	G < A	7.72E-05	0.51	0.81 [0.72–0.91]	2.88E-04	0.52	0.86 [0.71–1.03]	9.68E-02	0.20
2	rs78848661	185,999,116	*LOC105373782*	T < C	7.79E-05	0.66	0.75 [0.62–0.90]	2.71E-03	0.74	0.67 [0.50–0.90]	8.27E-03	0.29
10	rs587198	6,531,149	*PRKCQ*	C < T	7.87E-05	0.93	1.20 [1.08–1.34]	1.16E-03	0.67	1.22 [1.03–1.44]	2.47E-02	0.60
17	rs4471732	26,061,232	*NOS2*	G < A	8.38E-05	0.29	0.80 [0.71–0.89]	6.95E-05	0.19	0.91 [0.77–1.07]	2.62E-01	0.55
5	rs2255637	96,249,378	*ERAP1*	A < C	8.77E-05	0.48	1.18 [1.06–1.31]	3.13E-03	0.84	1.27 [1.06–1.51]	7.57E-03	0.16
17	rs12450521	26,083,392	*NOS2*	A < C	8.85E-05	0.72	0.77 [0.67–0.88]	1.51E-04	0.59	0.87 [0.72–1.05]	1.55E-01	0.84
15	rs4533267	100,786,271	*ADAMTS17*	A < G	9.63E-05	0.50	0.78 [0.69–0.88]	7.17E-05	0.85	0.91 [0.75–1.09]	3.00E-01	0.40
2	rs7349232	181,953,354	*UBE2E3*	T < C	9.84E-05	0.93	1.24 [1.09–1.41]	1.09E-03	0.50	1.25 [1.02–1.53]	3.39E-02	0.90
14	rs61981699	81,064,877	*CEP128*	T < C	9.88E-05	0.35	1.31 [1.13–1.52]	3.91E-04	0.15	1.23 [0.96–1.57]	9.68E-02	0.32
5	rs6874656	96,234,375	*ERAP1*	C < T	9.95E-05	0.46	1.18 [1.06–1.31]	3.20E-03	0.75	1.26 [1.06–1.50]	8.69E-03	0.15
5	rs251339	96,235,038	*ERAP1*	T < C	9.96E-05	0.72	1.19 [1.07–1.33]	1.64E-03	0.80	1.23 [1.03–1.46]	2.13E-02	0.27

BP, base-pair; CI, confidence interval; Chr, chromosome; GCA, giant cell arteritis; GRCh37, Genome Reference Consortium Human genome build 37; LVV, large vessel vasculitis; OR, odds ratio for the minor allele; Q, Cochran’s Q test P-value; SNP, single-nucleotide polymorphism; TAK, Takayasu’s arteritis.

**Table 2:** Functional annotations of the lead signal *IL12B* rs755374 and its proxies in the European populations of the 1000 genomes project.

SNP	Position in Chr5 (GRCh37)	Change	LD (r^2^/D’)	GRASP QTL hits	Functional annotations in immune cells			GWAS hits						
					H3K4me1	H3K4me3	DNAse peaks	Associated condition	P-value	OR	Population	Case/Control	Strategy	Ref
rs6871626	158,826,792	A < C	0.91/0.97	YES	NO	NO	NO	UC	1.11E-21	1.17	European	16,315/32,635	Meta GWAS	36
								IBD	1.00E-42	1.18	European	32,628/29,704	Meta GWAS + iChip	37
								AS	3.10E-02	1.12	Han Chinese	400/395	Candidate gene	38
								TAK	1.70E-13	1.75	Japanese	379/1,985	Exome GWAS	13
								Leprosy	3.95E-18	0.75	Chinese	4,971/5,503	Candidate gene	39
rs56167332	158,827,769	A < C	0.94/0.99	NO	NO	NO	NO	IBD	7.00E-50	1.17	European and Asian	42,950/53,536	GWAS + iChip	40
								CD	2.00E-41	1.19	European and Asian	22,575/46,693	GWAS + iChip	40
								UC	7.00E-27	1.15	European and Asian	20,417/52,230	GWAS + iChip	40
								TAK	2.18E-08	1.54	North American and Turkish	451/2,393	iChip	7
rs755374	158,829,294	A < G	NA	NO	YES	NO	NO	NA	NA	NA	NA	NA	NA	NA
rs4921492	158,832,277	A < C	0.90/0.99	NO	YES	YES	YES	Sarcoidosis	2.14E-09	1.20	European	1,726/5,482	iChip	41

AS, ankylosing spondylitis; CD, Crohn’s disease; Chr, chromosome; GWAS, genome-wide association study; GRASP, Genome-Wide Repository of Associations between SNPs and phenotypes; GRCh37, Genome Reference Consortium Human genome build 37; iChip, immunochip; IBD, Inflammatory bowel disease; LD, linkage disequilibrium; OR, odds ratio for the minor allele; QTL, quantitative trait loci; Ref, reference; SNP, single-nucleotide polymorphism; TAK, Takayasu’s arteritis; UC, ulcerative colitis.

**Table 3:** Genetic pleiotropy between giant cell arteritis and Takayasu’s arteritis using non-HLA data, HLA data only, and all Immunochip data.

Method	P-value		
	Non-HLA markers	HLA markers	All markers
REML	**5.00E-03**	5.00E-01	**6.00E-03**
PRS (GCA)	**3.53E-03**	2.68E-01	7.70E-02
PRS (TAK)	**3.60E-02**	6.97E-01	6.44E-01

GCA, giant cell arteritis; HLA, human leukocyte antigen; PRS, polygenic risk score; REML, restricted maximum likelihood; TAK, Takayasu’s arteritis.
