# Corrigendum: Analysis of the common genetic component of large-vessel vasculitides through a meta-Immunochip strategy

**DOI:** 10.1038/srep46012

**Published:** 2017-04-05

**Authors:** F. David Carmona, Patrick Coit, Güher Saruhan-Direskeneli, José Hernández-Rodríguez, María C. Cid, Roser Solans, Santos Castañeda, Augusto Vaglio, Haner Direskeneli, Peter A. Merkel, Luigi Boiardi, Carlo Salvarani, Miguel A. González-Gay, Javier Martín, Amr H. Sawalha, Spanish GCA Study Group, Agustín Martínez-Berriochoa, Ainhoa Unzurrunzaga, Ana Hidalgo-Conde, Ana Belén Madroñero Vuelta, Antonio Fernández-Nebro, M. Carmen Ordóñez-Cañizares, Benjamín Fernández-Gutiérrez, Luis Rodríguez-Rodríguez, Begoña Escalante, Begoña Marí-Alfonso, Bernardo Sopeña, Carmen Gómez-Vaquero, Enrique Raya, Elena Grau, José A. Román, Esther F. Vicente, Eugenio de Miguel, Francisco J. López-Longo, Lina Martínez, Inmaculada C. Morado, J. Bernardino Díaz-López, Luis Caminal-Montero, Aleida Martínez-Zapico, Javier Narváez, Jordi Monfort, Laura Tío, José A. Miranda-Filloy, Julio Sánchez-Martín, Juan J. Alegre-Sancho, Luis Sáez-Comet, Mercedes Pérez-Conesa, Marc Corbera-Bellalta, Marc Ramentol-Sintas, María Jesús García-Villanueva, Mercedes Guijarro Rojas, Norberto Ortego-Centeno, Raquel Ríos Fernández, José Luis Callejas, Olga Sanchez Pernaute, Patricia Fanlo Mateo, Ricardo Blanco, Sergio Prieto-González, Víctor Manuel Martínez-Taboada, Italian GCA Study Group, Alessandra Soriano, Claudio Lunardi, Davide Gianfreda, Daniele Santilli, Francesco Bonatti, Francesco Muratore, Giulia Pazzola, Olga Addimanda, Giacomo Emmi, Giuseppe A. Ramirez, Lorenzo Beretta, Marcello Govoni, Marco A. Cimmino, Turkish Takayasu Study Group, Ahmet Mesut Onat, Ayse Cefle, Ayten Yazici, Bünyamin Kısacık, Ediz Dalkilic, Emire Seyahi, Izzet Fresko, Ercan Tunc, Eren Erken, Hüseyin TE Ozer, Kenan Aksu, Gokhan Keser, Mehmet A. Ozturk, Muge Bıcakcıgil, Nurşen Duzgun, Omer Karadag, Sedat Kiraz, Ömer N. Pamuk, Servet Akar, Fatos Onen, Nurullah Akkoc, Sevil Kamali, Murat Inanc, Sibel P. Yentür, Sibel Z. Aydin, Fatma Alibaz-Oner, Timuçin Kaşifoğlu, Veli Cobankara, Zeynep Ozbalkan, Askin Ates, Yasar Karaaslan, Vasculitis Clinical Research Consortium, Simon Carette, Sharon A. Chung, David Cuthbertson, Lindsay J. Forbess, Gary S. Hoffman, Nader A. Khalidi, Curry L. Koening, Carol A. Langford, Carol A. McAlear, Kathleen McKinnon-Maksimowicz, Paul A. Monach, Larry Moreland, Christian Pagnoux, Philip Seo, Robert Spiera, Antoine G. Sreih, Kenneth J. Warrington, Steven R. Ytterberg

Scientific Reports 7: Article number: 4395310.1038/srep43953; published online: 03 09 2017; updated: 04 05 2017

In this Article, Javier Martin is incorrectly listed as being affiliated with “Departamento de Genética e Instituto de Biotecnología, Universidad de Granada, Granada 18016, Spain”. The correct affiliation is listed below:

Instituto de Parasitología y Biomedicina ‘López-Neyra’, IPBLN-CSIC, PTS Granada, Granada, Spain.

