# Supplementary material for: Analysis of the common genetic component of large-vessel vasculitides through a meta-Immunochip strategy
Source: Sci Rep. 2017 Mar 9;7:43953. doi: 10.1038/srep43953 (PMC5344032; doi:10.1038/srep43953)
Supplement: Supplementary Information [file srep43953-s1.pdf]

SUPPLEMENTARY FIGURES AND TABLES

Manuscript Title

Analysis of the common genetic component of large-vessel vasculitides through a meta-Immunochip strategy.

Authors

F. David Carmona, Patrick Coit, Güher Saruhan-Direskeneli, José Hernández-Rodríguez, María C. Cid, Roser Solans, Santos Castañeda, Augusto Vaglio, Haner Direskeneli, Peter A. Merkel, Luigi Boiardi, *Spanish GCA Study Group*, *Italian GCA Study Group*, *Turkish Takayasu Study Group*, *Vasculitis Clinical Research Consortium*, Carlo Salvarani, Miguel A. González-Gay, Javier Martín, Amr H. Sawalha.

Supplementary Figure S1. Manhattan plot representation of the results of the non-HLA region in (A) giant cell arteritis, (B) Takayasu's arteritis, and (C) the meta-analysis of both forms of vasculitis. The $\log_{10}$ of the P values are plotted against their physical chromosomal position. The red line represents the study-wide level of significance ($P < 1.13E-06$).

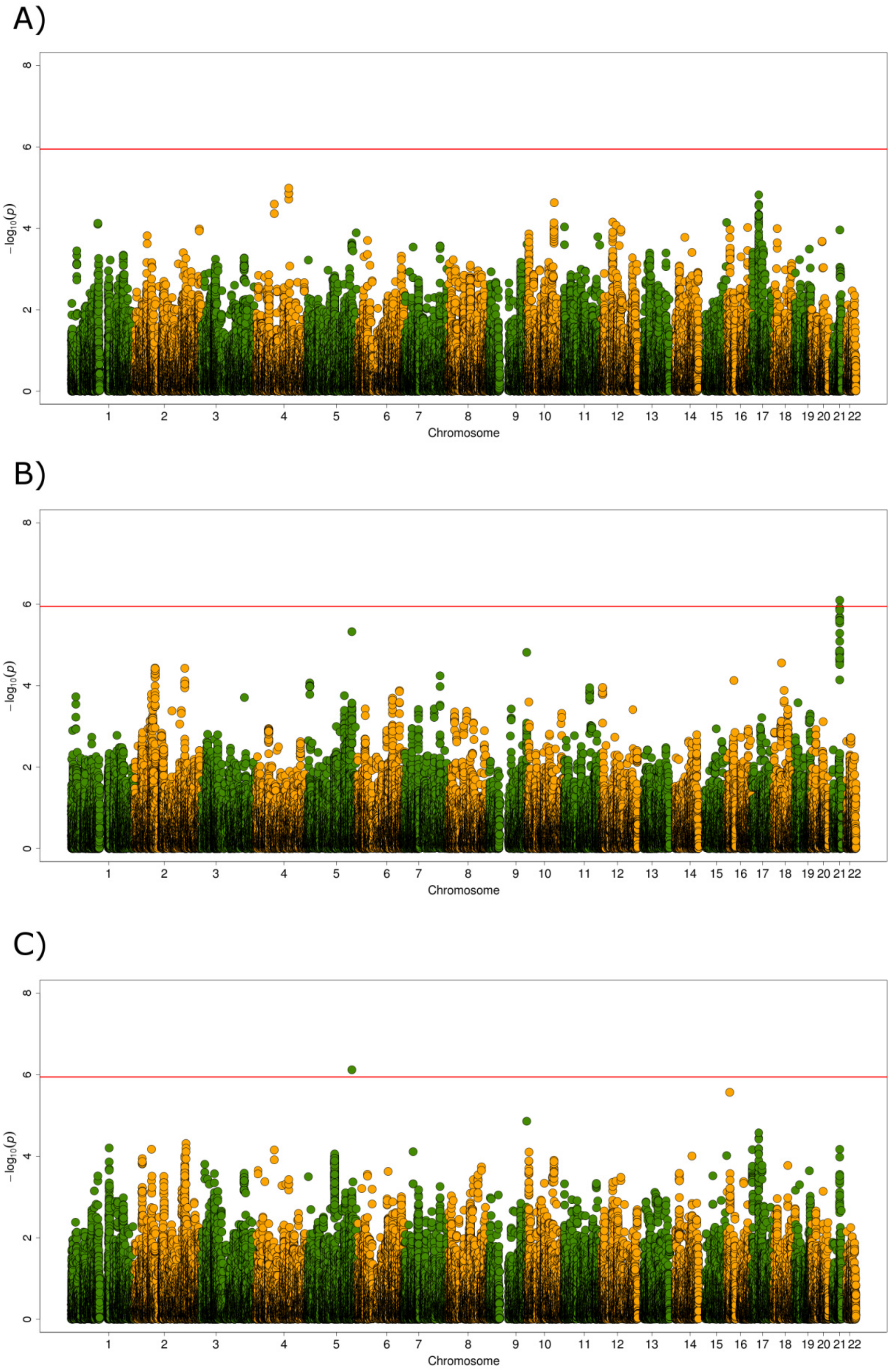

Supplementary Figure S2. Regional plot of the *IL12B* region showing the results of the meta-analysis between giant cell arteritis and Takayasu's arteritis. The lead signal rs755374 is highlighted.

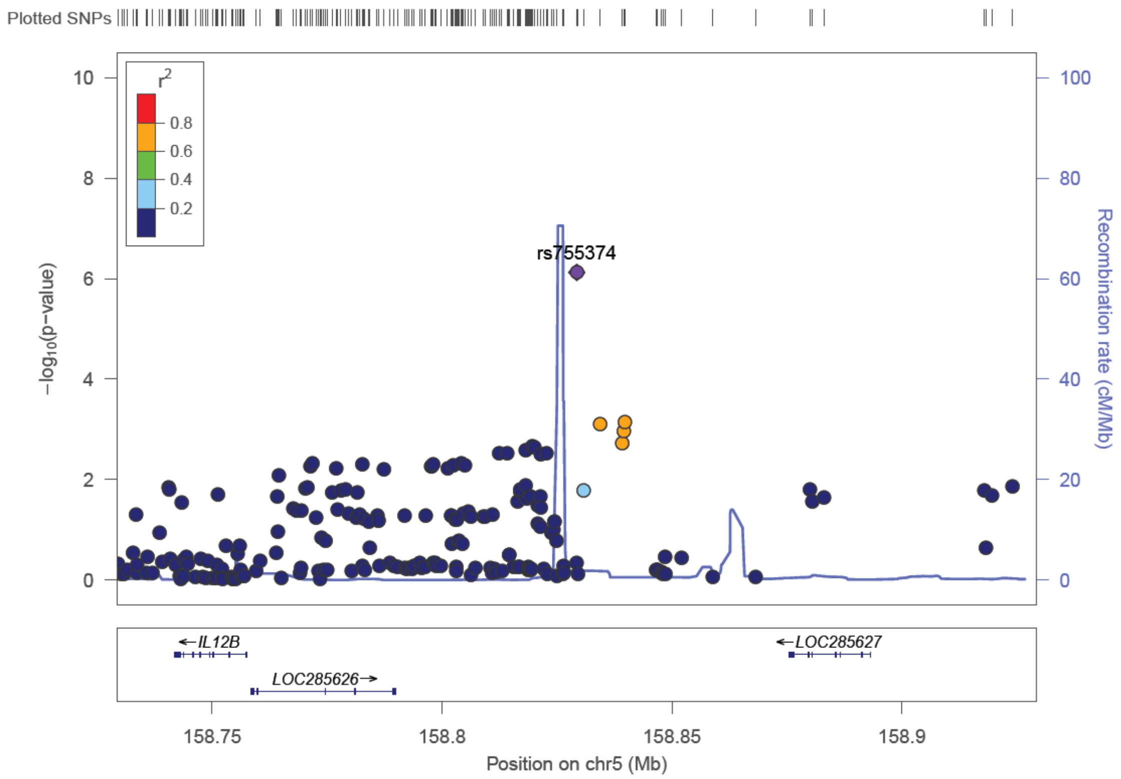

Supplementary Figure S3. Plot of the first and second principal components of the study cohorts, including Spain (red), Italy (blue), North America (green), and Turkey (grey). Controls and cases are represented with circles and squares, respectively.

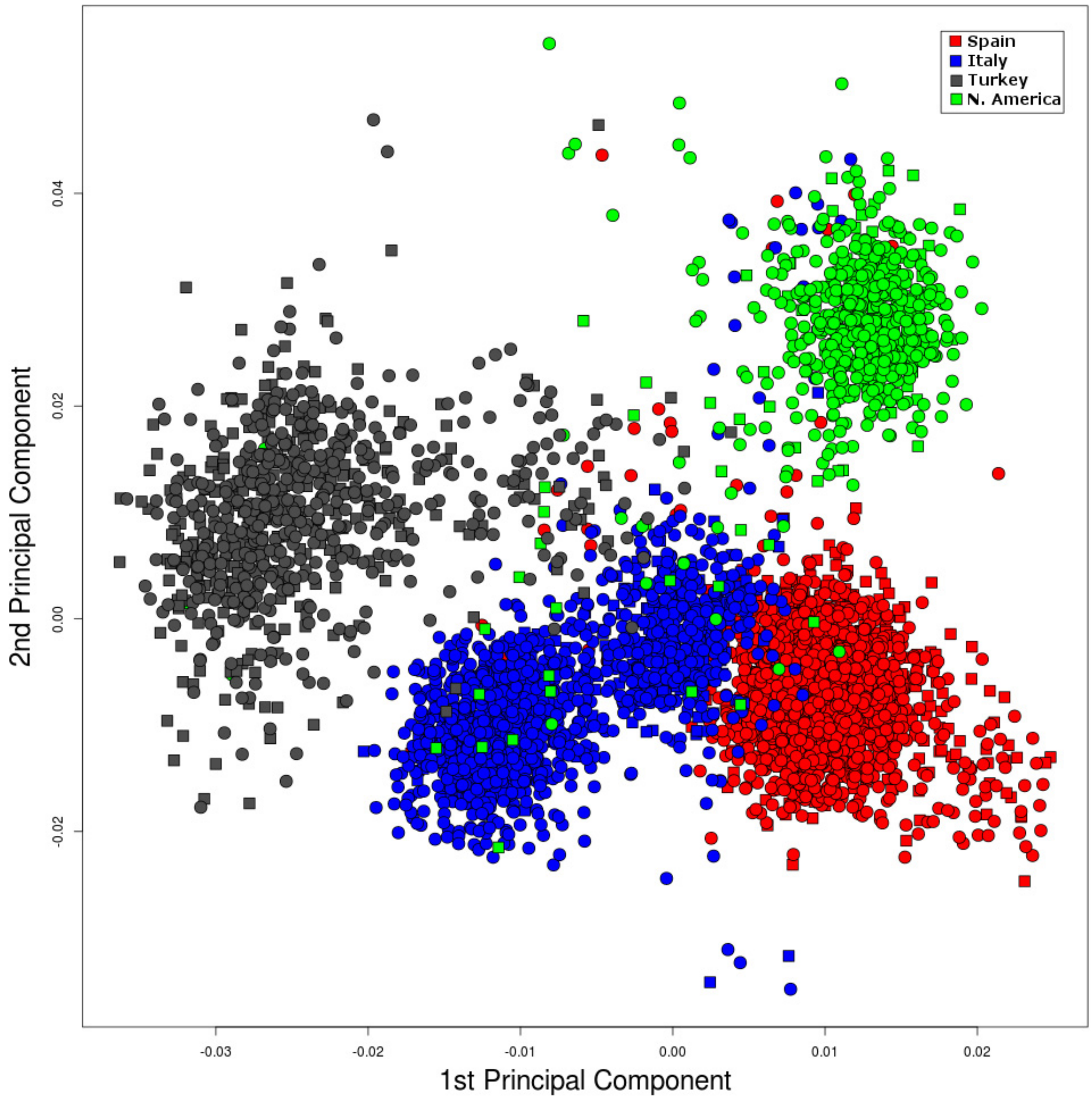

Supplementary Table S1. Suggestive shared signals (P < 1E-04) between giant cell arteritis (GCA) and Takayasu's arteritis (TAK) within the HLA region.

				META (GCA + TAK)		META (GCA)			META (TAK)			SPAIN (GCA)		ITALY (GCA)		NORTH AMERICA (TAK)		TURKEY (TAK)	
CHR	SNP	BP (GRCh37)	Change	P(R)	Q	OR [95% CI]	P	Q	OR [95% CI]	P	Q	OR [CI 95%]	P	OR [CI 95%]	P	OR [CI 95%]	P	OR [CI 95%]	P
6	rs9272105	32,599,999	G<A	3.74E-11	0.30	1.38 [1.23-1.53]	8.94E-09	0.36	1.57 [1.33-1.86]	1.78E-07	0.29	1.41 [1.25-1.60]	4.12E-08	1.25 [1.00-1.57]	5.09E-02	1.82 [1.32-2.51]	2.52E-04	1.48 [1.22-1.81]	1.08E-04
6	rs9271651	32,592,385	G<T	1.60E-09	0.29	1.34 [1.20-1.49]	1.25E-07	0.42	1.53 [1.29-1.82]	7.97E-07	0.24	1.37 [1.21-1.55]	5.57E-07	1.24 [0.99-1.54]	6.19E-02	1.81 [1.31-2.51]	3.53E-04	1.44 [1.18-1.76]	3.24E-04
6	rs9272218	32,602,238	C<T	1.83E-09	0.25	1.36 [1.22-1.52]	2.13E-08	0.31	1.57 [1.33-1.86]	1.69E-07	0.28	1.41 [1.24-1.59]	6.32E-08	1.23 [0.98-1.54]	7.50E-02	1.83 [1.33-2.53]	2.38E-04	1.48 [1.22-1.81]	1.06E-04
6	rs9271893	32,595,859	G<T	1.97E-09	0.29	1.32 [1.19-1.48]	3.19E-07	0.54	1.54 [1.30-1.82]	6.93E-07	0.26	1.35 [1.19-1.53]	1.79E-06	1.25 [1.00-1.56]	5.27E-02	1.80 [1.30-2.49]	3.84E-04	1.45 [1.19-1.77]	2.69E-04
6	rs9271858	32,595,223	A<G	2.49E-09	0.28	1.33 [1.20-1.49]	1.59E-07	0.43	1.54 [1.30-1.82]	7.52E-07	0.25	1.37 [1.21-1.55]	6.55E-07	1.23 [0.99-1.54]	6.43E-02	1.81 [1.31-2.51]	3.76E-04	1.45 [1.19-1.77]	2.85E-04
6	rs9272254	32,603,098	C<T	3.07E-09	0.24	1.36 [1.22-1.52]	2.56E-08	0.32	1.58 [1.33-1.87]	1.54E-07	0.26	1.40 [1.24-1.59]	8.03E-08	1.23 [0.98-1.55]	7.49E-02	1.85 [1.33-2.55]	2.18E-04	1.49 [1.22-1.81]	1.01E-04
6	rs9269081	32,441,100	A<C	6.40E-08	0.45	0.74 [0.66-0.84]	9.22E-07	0.15	0.80 [0.67-0.96]	1.70E-02	0.84	0.70 [0.61-0.81]	8.88E-07	0.85 [0.68-1.07]	1.60E-01	0.83 [0.60-1.15]	2.60E-01	0.80 [0.64-0.98]	3.44E-02
6	rs9268914	32,432,556	C<T	1.05E-07	0.47	0.75 [0.66-0.84]	1.58E-06	0.16	0.80 [0.67-0.96]	1.72E-02	0.83	0.71 [0.62-0.82]	1.43E-06	0.86 [0.69-1.07]	1.77E-01	0.83 [0.60-1.15]	2.63E-01	0.80 [0.64-0.98]	3.44E-02
6	rs9269027	32,437,466	A<T	2.55E-07	0.44	0.75 [0.66-0.84]	1.76E-06	0.17	0.82 [0.69-0.99]	3.38E-02	0.91	0.71 [0.62-0.82]	1.70E-06	0.86 [0.68-1.07]	1.73E-01	0.84 [0.60-1.16]	2.88E-01	0.82 [0.66-1.01]	6.56E-02
6	rs9263969	31,186,534	T<C	3.01E-07	0.75	0.77 [0.68-0.86]	1.16E-05	0.28	0.77 [0.64-0.94]	8.02E-03	0.90	0.80 [0.70-0.92]	1.46E-03	0.69 [0.55-0.87]	1.35E-03	0.79 [0.53-1.18]	2.52E-01	0.77 [0.62-0.95]	1.67E-02
6	rs2516006	32,650,268	C<T	4.73E-07	0.10	1.46 [1.29-1.65]	1.52E-09	0.79	1.61 [1.33-1.96]	1.27E-06	0.02	1.47 [1.28-1.69]	3.82E-08	1.41 [1.08-1.84]	1.18E-02	2.25 [1.60-3.15]	3.07E-06	1.38 [1.09-1.74]	7.81E-03
6	rs9263970	31,186,601	C<A	4.87E-07	0.71	0.77 [0.69-0.87]	1.84E-05	0.25	0.78 [0.64-0.94]	8.31E-03	0.88	0.81 [0.70-0.93]	2.22E-03	0.69 [0.55-0.86]	1.31E-03	0.80 [0.54-1.19]	2.63E-01	0.77 [0.62-0.95]	1.67E-02
6	rs10947210	31,417,742	C<T	1.07E-06	0.29	1.50 [1.20-1.87]	3.33E-04	0.36	1.99 [1.48-2.68]	5.42E-06	0.44	1.60 [1.23-2.07]	4.13E-04	1.27 [0.84-1.93]	2.63E-01	1.69 [1.01-2.83]	4.68E-02	2.17 [1.51-3.12]	3.13E-05
6	rs9405038	32,437,614	T<G	1.09E-06	0.11	1.60 [1.42-1.79]	6.65E-16	0.96	1.28 [1.06-1.54]	9.47E-03	0.13	1.60 [1.41-1.82]	9.43E-13	1.59 [1.25-2.02]	1.52E-04	1.56 [1.14-2.16]	6.14E-03	1.16 [0.92-1.45]	2.22E-01
6	rs9275205	32,657,560	C<T	1.11E-06	0.09	1.45 [1.29-1.64]	1.80E-09	0.92	1.58 [1.30-1.92]	3.78E-06	0.02	1.46 [1.27-1.67]	7.08E-08	1.44 [1.10-1.87]	7.52E-03	2.24 [1.59-3.14]	3.56E-06	1.34 [1.06-1.69]	1.59E-02
6	rs12174151	30,574,194	T<C	1.16E-06	0.40	1.51 [1.28-1.78]	7.64E-07	0.82	1.18 [0.92-1.53]	1.96E-01	0.53	1.50 [1.23-1.82]	5.82E-05	1.56 [1.15-2.11]	3.92E-03	1.31 [0.88-1.94]	1.88E-01	1.11 [0.79-1.55]	5.63E-01
6	rs3104407	32,682,452	G<A	3.53E-06	0.22	0.69 [0.62-0.77]	9.17E-11	0.88	0.86 [0.72-1.02]	8.19E-02	0.95	0.69 [0.61-0.79]	3.13E-08	0.68 [0.54-0.85]	7.45E-04	0.85 [0.62-1.16]	3.12E-01	0.86 [0.70-1.06]	1.57E-01
6	rs9275121	32,650,185	G<A	3.79E-06	0.10	1.41 [1.25-1.58]	2.12E-08	0.82	1.52 [1.26-1.83]	1.30E-05	0.02	1.42 [1.24-1.62]	4.58E-07	1.37 [1.06-1.76]	1.47E-02	2.11 [1.52-2.92]	7.52E-06	1.29 [1.03-1.63]	2.96E-02
6	rs9275117	32,649,965	T<C	4.20E-06	0.09	1.42 [1.26-1.60]	1.06E-08	0.78	1.51 [1.25-1.83]	1.53E-05	0.01	1.43 [1.25-1.64]	2.25E-07	1.37 [1.07-1.77]	1.40E-02	2.12 [1.53-2.93]	6.76E-06	1.28 [1.02-1.61]	3.41E-02
6	rs16868789	33,072,598	G<A	5.37E-06	0.42	1.62 [1.23-2.13]	6.00E-04	0.35	2.28 [1.38-3.76]	1.33E-03	0.44	1.72 [1.27-2.34]	4.72E-04	1.24 [0.66-2.31]	5.09E-01	1.71 [0.70-4.17]	2.42E-01	2.60 [1.42-4.78]	2.03E-03
6	rs17221073	33,048,371	C<G	5.60E-06	0.43	1.60 [1.22-2.10]	7.17E-04	0.36	2.30 [1.40-3.79]	1.04E-03	0.56	1.70 [1.26-2.30]	5.76E-04	1.23 [0.66-2.30]	5.17E-01	1.79 [0.68-4.73]	2.41E-01	2.52 [1.41-4.50]	1.82E-03
6	rs12665339	30,601,232	G<A	5.86E-06	0.36	1.52 [1.29-1.79]	7.09E-07	0.91	1.17 [0.91-1.51]	2.20E-01	0.52	1.51 [1.24-1.84]	4.15E-05	1.54 [1.14-2.08]	5.24E-03	1.30 [0.87-1.93]	1.99E-01	1.09 [0.79-1.52]	5.96E-01
6	rs9275134	32,650,612	G<C	5.98E-06	0.08	1.41 [1.25-1.59]	1.67E-08	0.83	1.52 [1.26-1.83]	1.33E-05	0.01	1.42 [1.24-1.62]	3.84E-07	1.38 [1.07-1.77]	1.30E-02	2.13 [1.54-2.95]	5.52E-06	1.28 [1.02-1.61]	3.31E-02
6	rs9263985	31,190,538	A<G	6.04E-06	0.82	0.83 [0.74-0.92]	7.22E-04	0.72	0.76 [0.63-0.90]	1.77E-03	0.81	0.84 [0.74-0.95]	6.61E-03	0.80 [0.65-0.99]	4.07E-02	0.73 [0.52-1.02]	6.72E-02	0.77 [0.62-0.94]	1.09E-02
6	rs9275132	32,650,500	A<G	6.24E-06	0.08	1.41 [1.25-1.58]	1.87E-08	0.84	1.52 [1.26-1.83]	1.33E-05	0.01	1.42 [1.24-1.62]	4.29E-07	1.38 [1.07-1.77]	1.30E-02	2.13 [1.54-2.95]	5.52E-06	1.28 [1.02-1.61]	3.31E-02
6	rs2856692	32,653,385	G<T	6.87E-06	0.08	1.41 [1.25-1.58]	1.82E-08	0.79	1.52 [1.26-1.83]	1.29E-05	0.01	1.42 [1.24-1.62]	3.57E-07	1.37 [1.06-1.76]	1.53E-02	2.13 [1.54-2.95]	5.39E-06	1.28 [1.02-1.61]	3.23E-02
6	rs2856683	32,655,218	G<T	7.91E-06	0.08	1.40 [1.25-1.58]	2.65E-08	0.89	1.51 [1.25-1.82]	1.79E-05	0.01	1.41 [1.23-1.61]	6.94E-07	1.38 [1.07-1.78]	1.19E-02	2.12 [1.53-2.94]	6.08E-06	1.27 [1.01-1.60]	3.83E-02
6	rs1799724	31,542,482	T<C	9.95E-06	0.30	0.75 [0.63-0.90]	2.11E-03	0.68	0.59 [0.47-0.75]	1.98E-05	0.33	0.73 [0.58-0.92]	7.17E-03	0.79 [0.59-1.06]	1.22E-01	0.76 [0.44-1.33]	3.34E-01	0.56 [0.43-0.73]	1.95E-05
6	rs2856688	32,654,640	T<G	1.01E-05	0.07	1.41 [1.25-1.58]	2.11E-08	0.88	1.52 [1.26-1.83]	1.37E-05	0.01	1.41 [1.23-1.62]	5.60E-07	1.38 [1.08-1.78]	1.17E-02	2.16 [1.55-2.99]	4.34E-06	1.28 [1.02-1.60]	3.58E-02
6	rs58854385	33,049,015	C<T	1.07E-05	0.37	1.60 [1.22-2.10]	7.32E-04	0.35	2.36 [1.44-3.88]	7.04E-04	0.52	1.70 [1.26-2.30]	5.64E-04	1.22 [0.65-2.28]	5.29E-01	1.79 [0.68-4.73]	2.41E-01	2.60 [1.46-4.64]	1.18E-03
6	rs57937516	33,049,131	A<G	1.07E-05	0.37	1.60 [1.22-2.10]	7.32E-04	0.35	2.36 [1.44-3.88]	7.04E-04	0.52	1.70 [1.26-2.30]	5.64E-04	1.22 [0.65-2.28]	5.29E-01	1.79 [0.68-4.73]	2.41E-01	2.60 [1.46-4.64]	1.18E-03
6	rs58467645	33,049,201	G<A	1.07E-05	0.37	1.60 [1.22-2.10]	7.32E-04	0.35	2.36 [1.44-3.88]	7.04E-04	0.52	1.70 [1.26-2.30]	5.64E-04	1.22 [0.65-2.28]	5.29E-01	1.79 [0.68-4.73]	2.41E-01	2.60 [1.46-4.64]	1.18E-03
6	rs4947342	32,653,070	A<G	1.18E-05	0.07	1.41 [1.25-1.58]	2.19E-08	0.79	1.52 [1.26-1.83]	1.29E-05	0.01	1.42 [1.24-1.62]	4.19E-07	1.36 [1.06-1.76]	1.60E-02	2.16 [1.56-3.00]	4.31E-06	1.28 [1.02-1.61]	3.41E-02

6	rs3918154	32,943,752	G<A	1.19E-05	0.38	1.52 [1.16-1.99]	2.15E-03	0.46	2.03 [1.36-3.05]	5.92E-04	0.28	1.63 [1.18-2.26]	3.21E-03	1.32 [0.82-2.12]	2.59E-01	1.30 [0.52-3.23]	5.75E-01	2.27 [1.45-3.57]	3.76E-04
6	rs56952336	33,040,691	A<G	1.26E-05	0.43	1.58 [1.20-2.09]	1.20E-03	0.31	2.29 [1.37-3.80]	1.46E-03	0.67	1.70 [1.25-2.31]	7.71E-04	1.17 [0.61-2.23]	6.37E-01	1.91 [0.71-5.13]	2.02E-01	2.44 [1.35-4.42]	3.24E-03
6	rs116050038	33,050,455	G<A	1.33E-05	0.35	1.66 [1.26-2.19]	2.97E-04	0.30	2.39 [1.45-3.94]	6.65E-04	0.40	1.79 [1.31-2.43]	2.16E-04	1.24 [0.66-2.32]	5.01E-01	1.68 [0.64-4.39]	2.94E-01	2.72 [1.52-4.90]	8.20E-04
6	rs3131063	30,763,756	A<G	1.33E-05	0.79	1.21 [1.08-1.35]	8.85E-04	0.60	1.29 [1.08-1.53]	4.03E-03	0.51	1.23 [1.08-1.40]	1.72E-03	1.15 [0.92-1.43]	2.18E-01	1.41 [1.03-1.92]	3.27E-02	1.24 [1.01-1.52]	4.20E-02
6	rs61406966	33,049,123	A<G	1.61E-05	0.37	1.58 [1.21-2.08]	8.94E-04	0.36	2.36 [1.44-3.88]	7.04E-04	0.52	1.68 [1.25-2.28]	7.06E-04	1.22 [0.65-2.28]	5.32E-01	1.79 [0.68-4.73]	2.41E-01	2.60 [1.46-4.64]	1.18E-03
6	rs6927229	28,417,737	A<T	1.63E-05	0.66	0.80 [0.71-0.89]	1.08E-04	0.42	0.83 [0.69-1.00]	5.39E-02	0.37	0.78 [0.68-0.89]	1.51E-04	0.87 [0.68-1.11]	2.60E-01	0.73 [0.52-1.03]	6.87E-02	0.88 [0.70-1.11]	2.72E-01
6	rs6922111	28,325,308	T<C	1.71E-05	0.98	0.78 [0.68-0.89]	3.41E-04	0.84	0.74 [0.58-0.95]	1.64E-02	0.89	0.77 [0.66-0.90]	1.03E-03	0.80 [0.59-1.08]	1.47E-01	0.73 [0.48-1.11]	1.35E-01	0.75 [0.56-1.01]	5.98E-02
6	rs9366801	32,440,276	T<C	1.83E-05	0.06	1.59 [1.42-1.78]	1.46E-15	0.96	1.25 [1.04-1.51]	1.62E-02	0.08	1.58 [1.39-1.80]	2.36E-12	1.59 [1.25-2.02]	1.36E-04	1.58 [1.15-2.17]	5.08E-03	1.12 [0.89-1.40]	3.39E-01
6	rs73741627	33,047,646	C<T	1.85E-05	0.36	1.55 [1.19-2.02]	1.02E-03	0.38	2.30 [1.44-3.69]	5.27E-04	0.54	1.65 [1.23-2.21]	8.29E-04	1.22 [0.66-2.23]	5.28E-01	1.84 [0.79-4.31]	1.58E-01	2.54 [1.44-4.48]	1.25E-03
6	rs145187462	33,069,495	G<C	1.86E-05	0.36	1.62 [1.23-2.13]	5.45E-04	0.34	2.27 [1.38-3.75]	1.29E-03	0.33	1.73 [1.28-2.35]	4.28E-04	1.24 [0.66-2.31]	5.09E-01	1.53 [0.59-3.94]	3.79E-01	2.65 [1.47-4.79]	1.18E-03
6	rs11752919	28,403,603	C<T	2.06E-05	0.67	0.80 [0.71-0.89]	9.68E-05	0.42	0.84 [0.70-1.02]	7.45E-02	0.41	0.78 [0.68-0.88]	1.39E-04	0.87 [0.68-1.11]	2.52E-01	0.75 [0.53-1.05]	9.28E-02	0.89 [0.71-1.12]	3.09E-01
6	rs9268923	32,432,835	T<C	2.06E-05	0.05	1.58 [1.41-1.77]	1.45E-15	0.95	1.26 [1.05-1.51]	1.49E-02	0.07	1.58 [1.39-1.79]	2.49E-12	1.59 [1.26-2.03]	1.32E-04	1.60 [1.16-2.20]	3.89E-03	1.11 [0.89-1.40]	3.48E-01
6	rs1034322	30,368,854	T<C	2.19E-05	0.80	1.35 [1.16-1.58]	1.33E-04	0.72	1.26 [0.99-1.61]	5.64E-02	0.41	1.38 [1.15-1.66]	6.19E-04	1.30 [0.97-1.74]	8.41E-02	1.44 [0.98-2.11]	6.65E-02	1.17 [0.86-1.59]	3.30E-01
6	rs9368726	32,438,542	C<T	2.20E-05	0.06	1.57 [1.41-1.76]	2.78E-15	0.99	1.26 [1.05-1.51]	1.47E-02	0.07	1.57 [1.39-1.79]	3.21E-12	1.58 [1.24-2.01]	1.97E-04	1.60 [1.17-2.20]	3.61E-03	1.11 [0.89-1.39]	3.59E-01
6	rs9262192	30,745,454	C<T	2.29E-05	0.90	0.54 [0.40-0.73]	4.80E-05	0.69	0.63 [0.31-1.29]	2.10E-01	0.60	0.53 [0.38-0.73]	9.51E-05	0.62 [0.29-1.35]	2.28E-01	0.44 [0.09-2.09]	3.00E-01	0.70 [0.31-1.56]	3.82E-01
6	rs1592415	30,377,906	G<T	2.62E-05	0.73	1.35 [1.16-1.58]	1.58E-04	0.72	1.26 [0.99-1.61]	5.75E-02	0.33	1.38 [1.15-1.66]	6.81E-04	1.29 [0.96-1.73]	9.00E-02	1.47 [1.00-2.17]	5.09E-02	1.15 [0.84-1.56]	3.80E-01
6	rs4711217	30,379,835	A<C	2.91E-05	0.74	1.35 [1.15-1.58]	1.89E-04	0.70	1.27 [1.00-1.62]	5.39E-02	0.33	1.38 [1.14-1.66]	7.70E-04	1.29 [0.96-1.73]	9.71E-02	1.47 [1.00-2.17]	5.00E-02	1.15 [0.85-1.57]	3.69E-01
6	rs9405108	32,438,648	T<C	3.11E-05	0.05	1.58 [1.41-1.76]	2.20E-15	0.92	1.25 [1.04-1.50]	1.69E-02	0.06	1.57 [1.38-1.79]	3.81E-12	1.59 [1.26-2.03]	1.32E-04	1.60 [1.16-2.20]	3.80E-03	1.11 [0.88-1.39]	3.81E-01
6	rs6903535	28,417,222	G<A	3.11E-05	0.67	0.80 [0.72-0.90]	1.72E-04	0.43	0.84 [0.69-1.01]	6.63E-02	0.37	0.78 [0.69-0.89]	2.33E-04	0.87 [0.69-1.11]	2.76E-01	0.74 [0.53-1.04]	7.79E-02	0.89 [0.71-1.12]	3.04E-01
6	rs34039931	30,429,355	G<A	3.15E-05	0.47	1.39 [1.18-1.64]	6.89E-05	0.81	1.21 [0.95-1.54]	1.24E-01	0.21	1.41 [1.16-1.71]	5.20E-04	1.35 [1.00-1.82]	4.91E-02	1.46 [1.00-2.14]	5.21E-02	1.07 [0.78-1.46]	6.88E-01
6	rs9263993	31,192,766	T<C	3.19E-05	0.61	0.83 [0.73-0.94]	3.19E-03	0.59	0.71 [0.57-0.88]	1.47E-03	0.93	0.84 [0.73-0.98]	2.16E-02	0.78 [0.60-1.00]	5.41E-02	0.72 [0.48-1.10]	1.25E-01	0.71 [0.55-0.90]	5.32E-03
6	rs9268853	32,429,643	C<T	3.28E-05	0.04	1.59 [1.42-1.77]	1.01E-15	0.87	1.25 [1.04-1.50]	1.71E-02	0.06	1.58 [1.39-1.79]	2.59E-12	1.61 [1.27-2.05]	8.35E-05	1.60 [1.16-2.20]	3.89E-03	1.11 [0.88-1.39]	3.81E-01
6	rs71565340	32,452,506	T<C	3.37E-05	0.05	1.58 [1.42-1.77]	1.44E-15	0.96	1.25 [1.04-1.51]	1.64E-02	0.06	1.59 [1.40-1.80]	1.60E-12	1.58 [1.24-2.00]	2.10E-04	1.60 [1.17-2.20]	3.72E-03	1.11 [0.88-1.39]	3.80E-01
6	rs2395185	32,433,167	T<G	3.39E-05	0.05	1.58 [1.41-1.77]	1.45E-15	0.95	1.25 [1.04-1.51]	1.65E-02	0.06	1.58 [1.39-1.79]	2.49E-12	1.59 [1.26-2.03]	1.32E-04	1.61 [1.17-2.21]	3.58E-03	1.11 [0.88-1.39]	3.81E-01
6	rs9405112	32,445,600	A<G	3.43E-05	0.05	1.58 [1.41-1.76]	2.31E-15	0.91	1.25 [1.04-1.50]	1.70E-02	0.06	1.57 [1.38-1.79]	3.85E-12	1.60 [1.26-2.03]	1.31E-04	1.60 [1.17-2.20]	3.61E-03	1.10 [0.88-1.38]	3.92E-01
6	rs28654725	32,435,542	G<A	3.45E-05	0.05	1.57 [1.41-1.76]	3.54E-15	0.93	1.25 [1.04-1.50]	1.77E-02	0.06	1.57 [1.38-1.78]	5.26E-12	1.59 [1.25-2.02]	1.55E-04	1.60 [1.16-2.20]	3.78E-03	1.10 [0.88-1.38]	3.96E-01
6	rs28895224	32,435,556	G<A	3.45E-05	0.05	1.57 [1.40-1.76]	3.75E-15	0.95	1.25 [1.04-1.50]	1.77E-02	0.06	1.57 [1.38-1.78]	5.26E-12	1.58 [1.25-2.01]	1.65E-04	1.60 [1.16-2.20]	3.78E-03	1.10 [0.88-1.38]	3.96E-01
6	rs9268969	32,434,349	T<C	3.46E-05	0.05	1.58 [1.41-1.77]	1.54E-15	0.97	1.25 [1.04-1.50]	1.76E-02	0.07	1.58 [1.39-1.80]	2.34E-12	1.59 [1.25-2.02]	1.52E-04	1.59 [1.16-2.19]	4.08E-03	1.11 [0.88-1.39]	3.81E-01
6	rs9268889	32,431,886	G<A	3.47E-05	0.05	1.58 [1.41-1.76]	2.78E-15	0.99	1.25 [1.04-1.50]	1.84E-02	0.07	1.58 [1.39-1.79]	3.53E-12	1.58 [1.24-2.01]	1.85E-04	1.59 [1.16-2.19]	4.33E-03	1.11 [0.88-1.39]	3.88E-01
6	rs34720986	32,593,270	C<G	3.61E-05	0.43	0.68 [0.56-0.83]	1.28E-04	0.16	0.77 [0.57-1.04]	9.13E-02	0.54	0.62 [0.49-0.79]	6.51E-05	0.86 [0.58-1.25]	4.21E-01	0.88 [0.52-1.50]	6.41E-01	0.72 [0.50-1.04]	8.24E-02
6	rs1694111	32,648,753	T<C	3.79E-05	0.43	1.26 [1.08-1.46]	3.76E-03	0.93	1.62 [1.22-2.16]	9.25E-04	0.53	1.25 [1.05-1.49]	1.15E-02	1.27 [0.91-1.78]	1.56E-01	1.44 [0.89-2.32]	1.37E-01	1.74 [1.21-2.49]	2.51E-03
6	rs114305335	33,059,947	T<C	3.85E-05	0.33	1.64 [1.25-2.15]	4.02E-04	0.34	2.32 [1.41-3.83]	9.26E-04	0.31	1.75 [1.29-2.37]	3.11E-04	1.24 [0.66-2.32]	4.98E-01	1.53 [0.59-3.94]	3.79E-01	2.73 [1.52-4.91]	8.01E-04
6	rs769178	31,547,514	T<G	3.91E-05	0.27	0.76 [0.63-0.91]	2.68E-03	0.44	0.60 [0.47-0.76]	3.41E-05	0.33	0.72 [0.57-0.90]	4.61E-03	0.83 [0.62-1.11]	2.07E-01	0.77 [0.44-1.35]	3.61E-01	0.57 [0.43-0.74]	3.17E-05
6	rs34521355	30,429,330	G<T	3.96E-05	0.51	1.39 [1.18-1.63]	7.19E-05	0.80	1.20 [0.94-1.53]	1.45E-01	0.26	1.41 [1.16-1.71]	5.20E-04	1.34 [1.00-1.81]	5.12E-02	1.43 [0.97-2.10]	7.25E-02	1.07 [0.78-1.46]	6.68E-01
6	rs71565333	32,451,902	T<C	3.99E-05	0.05	1.58 [1.41-1.77]	2.19E-15	0.95	1.24 [1.03-1.49]	2.20E-02	0.08	1.58 [1.39-1.80]	3.58E-12	1.59 [1.26-2.02]	1.34E-04	1.57 [1.14-2.17]	5.96E-03	1.11 [0.88-1.39]	3.86E-01
6	rs62405565	32,452,281	G<C	4.05E-05	0.05	1.58 [1.41-1.76]	2.62E-15	0.92	1.25 [1.04-1.50]	1.81E-02	0.06	1.57 [1.38-1.78]	4.47E-12	1.59 [1.26-2.02]	1.34E-04	1.60 [1.17-2.20]	3.66E-03	1.10 [0.88-1.38]	4.02E-01
6	rs7747529	32,431,124	A<G	4.10E-05	0.05	1.58 [1.41-1.76]	2.41E-15	0.92	1.25 [1.04-1.50]	1.83E-02	0.06	1.57 [1.38-1.79]	3.87E-12	1.59 [1.25-2.02]	1.35E-04	1.60 [1.16-2.20]	3.75E-03	1.10 [0.88-1.38]	4.02E-01

6	rs9268860	32,429,825	T<C	4.11E-05	0.04	1.58 [1.41-1.77]	1.42E-15	0.84	1.25 [1.04-1.50]	1.88E-02	0.06	1.57 [1.38-1.79]	3.58E-12	1.61 [1.27-2.05]	8.35E-05	1.60 [1.16-2.20]	3.94E-03	1.10 [0.88-1.38]	4.02E-01
6	rs9268858	32,429,758	C<T	4.11E-05	0.04	1.58 [1.41-1.77]	1.43E-15	0.84	1.25 [1.04-1.50]	1.88E-02	0.06	1.57 [1.38-1.79]	3.69E-12	1.61 [1.27-2.05]	8.35E-05	1.60 [1.16-2.20]	3.94E-03	1.10 [0.88-1.38]	4.02E-01
6	rs9268884	32,431,640	A<T	4.16E-05	0.05	1.57 [1.40-1.76]	4.67E-15	0.99	1.25 [1.04-1.50]	1.84E-02	0.06	1.57 [1.38-1.78]	4.79E-12	1.57 [1.24-2.00]	2.21E-04	1.60 [1.16-2.20]	3.81E-03	1.10 [0.88-1.38]	4.02E-01
6	rs79452641	32,451,288	C<G	4.16E-05	0.04	1.58 [1.41-1.77]	2.29E-15	0.92	1.25 [1.04-1.50]	1.83E-02	0.06	1.57 [1.38-1.79]	3.92E-12	1.60 [1.26-2.03]	1.30E-04	1.60 [1.16-2.20]	3.79E-03	1.10 [0.88-1.38]	4.02E-01
6	rs77777792	32,451,289	T<A	4.16E-05	0.04	1.58 [1.41-1.77]	2.29E-15	0.92	1.25 [1.04-1.50]	1.83E-02	0.06	1.57 [1.38-1.79]	3.92E-12	1.60 [1.26-2.03]	1.30E-04	1.60 [1.16-2.20]	3.79E-03	1.10 [0.88-1.38]	4.02E-01
6	rs12197312	32,442,180	C<T	4.16E-05	0.04	1.58 [1.41-1.77]	2.00E-15	0.92	1.25 [1.04-1.50]	1.79E-02	0.06	1.57 [1.39-1.79]	3.39E-12	1.60 [1.26-2.03]	1.31E-04	1.60 [1.17-2.20]	3.61E-03	1.10 [0.88-1.38]	4.02E-01
6	rs9268864	32,430,326	A<T	4.16E-05	0.04	1.58 [1.41-1.77]	1.32E-15	0.85	1.25 [1.04-1.50]	1.92E-02	0.06	1.57 [1.38-1.79]	3.45E-12	1.61 [1.27-2.05]	8.26E-05	1.59 [1.16-2.19]	4.08E-03	1.10 [0.88-1.38]	4.02E-01
6	rs9268905	32,432,077	C<G	4.18E-05	0.05	1.58 [1.41-1.76]	2.14E-15	0.92	1.25 [1.04-1.50]	1.90E-02	0.06	1.57 [1.38-1.79]	3.61E-12	1.60 [1.26-2.03]	1.31E-04	1.60 [1.16-2.19]	4.00E-03	1.10 [0.88-1.38]	4.02E-01
6	rs9348886	32,441,008	T<C	4.18E-05	0.04	1.58 [1.41-1.77]	2.04E-15	0.92	1.25 [1.04-1.50]	1.81E-02	0.06	1.57 [1.39-1.79]	3.43E-12	1.59 [1.26-2.03]	1.32E-04	1.60 [1.17-2.20]	3.66E-03	1.10 [0.88-1.38]	4.02E-01
6	rs28895234	32,437,050	G<A	4.19E-05	0.04	1.58 [1.41-1.77]	1.85E-15	0.92	1.25 [1.04-1.50]	1.77E-02	0.06	1.57 [1.39-1.79]	3.11E-12	1.60 [1.26-2.03]	1.30E-04	1.60 [1.17-2.20]	3.55E-03	1.10 [0.88-1.38]	4.02E-01
6	rs9368725	32,438,159	T<C	4.19E-05	0.04	1.58 [1.41-1.77]	1.86E-15	0.92	1.25 [1.04-1.50]	1.77E-02	0.06	1.57 [1.39-1.79]	3.21E-12	1.60 [1.26-2.03]	1.30E-04	1.60 [1.17-2.20]	3.55E-03	1.10 [0.88-1.38]	4.02E-01
6	rs9268906	32,432,181	G<A	4.19E-05	0.05	1.58 [1.41-1.76]	2.14E-15	0.92	1.25 [1.04-1.50]	1.92E-02	0.06	1.57 [1.38-1.79]	3.61E-12	1.60 [1.26-2.03]	1.31E-04	1.59 [1.16-2.19]	4.07E-03	1.10 [0.88-1.38]	4.02E-01
6	rs9380306	32,438,298	G<A	4.20E-05	0.04	1.58 [1.41-1.77]	1.86E-15	0.92	1.25 [1.04-1.50]	1.79E-02	0.06	1.57 [1.39-1.79]	3.21E-12	1.60 [1.26-2.03]	1.30E-04	1.60 [1.17-2.20]	3.61E-03	1.10 [0.88-1.38]	4.02E-01
6	rs9391877	32,438,867	T<G	4.22E-05	0.04	1.58 [1.41-1.77]	1.67E-15	0.90	1.25 [1.04-1.50]	1.79E-02	0.06	1.57 [1.39-1.79]	3.11E-12	1.60 [1.26-2.03]	1.15E-04	1.60 [1.17-2.20]	3.61E-03	1.10 [0.88-1.38]	4.02E-01
6	rs9405109	32,439,029	C<T	4.22E-05	0.04	1.58 [1.41-1.77]	1.67E-15	0.90	1.25 [1.04-1.50]	1.79E-02	0.06	1.57 [1.39-1.79]	3.11E-12	1.60 [1.26-2.03]	1.15E-04	1.60 [1.17-2.20]	3.61E-03	1.10 [0.88-1.38]	4.02E-01
6	rs9391781	32,434,850	C<G	4.22E-05	0.04	1.58 [1.41-1.77]	2.00E-15	0.92	1.25 [1.04-1.50]	1.89E-02	0.06	1.57 [1.39-1.79]	3.36E-12	1.60 [1.26-2.03]	1.30E-04	1.60 [1.16-2.19]	3.97E-03	1.10 [0.88-1.38]	4.02E-01
6	rs28895235	32,437,160	A<T	4.24E-05	0.04	1.58 [1.41-1.77]	1.53E-15	0.91	1.25 [1.04-1.50]	1.77E-02	0.06	1.58 [1.39-1.79]	3.04E-12	1.60 [1.26-2.03]	1.14E-04	1.60 [1.17-2.20]	3.55E-03	1.10 [0.88-1.38]	4.02E-01
6	rs9268965	32,434,240	A<G	4.26E-05	0.04	1.58 [1.41-1.77]	1.89E-15	0.93	1.25 [1.04-1.50]	1.89E-02	0.06	1.57 [1.39-1.79]	3.12E-12	1.59 [1.26-2.03]	1.32E-04	1.60 [1.16-2.19]	3.97E-03	1.10 [0.88-1.38]	4.02E-01
6	rs9268970	32,434,361	A<C	4.26E-05	0.04	1.58 [1.41-1.77]	1.89E-15	0.93	1.25 [1.04-1.50]	1.89E-02	0.06	1.57 [1.39-1.79]	3.12E-12	1.59 [1.26-2.03]	1.32E-04	1.60 [1.16-2.19]	3.97E-03	1.10 [0.88-1.38]	4.02E-01
6	rs35372060	32,447,667	T<C	4.31E-05	0.05	1.57 [1.40-1.75]	7.46E-15	0.85	1.24 [1.03-1.49]	2.37E-02	0.07	1.56 [1.37-1.77]	1.24E-11	1.60 [1.26-2.03]	1.32E-04	1.58 [1.14-2.19]	5.58E-03	1.10 [0.88-1.38]	4.09E-01
6	rs33950405	30,396,320	T<A	4.35E-05	0.56	1.35 [1.15-1.58]	1.92E-04	0.59	1.24 [0.98-1.59]	7.80E-02	0.22	1.39 [1.15-1.67]	5.60E-04	1.26 [0.93-1.70]	1.31E-01	1.50 [1.02-2.21]	3.96E-02	1.10 [0.81-1.51]	5.48E-01
6	rs916571	30,201,955	A<G	4.37E-05	0.90	1.20 [1.08-1.35]	1.14E-03	0.87	1.26 [1.05-1.51]	1.21E-02	0.53	1.20 [1.05-1.36]	6.35E-03	1.22 [0.98-1.53]	7.59E-02	1.16 [0.84-1.60]	3.73E-01	1.31 [1.05-1.63]	1.52E-02
6	rs35508382	32,593,144	G<A	4.49E-05	0.45	0.68 [0.56-0.83]	1.62E-04	0.17	0.77 [0.57-1.04]	9.06E-02	0.59	0.62 [0.49-0.79]	8.04E-05	0.86 [0.58-1.26]	4.40E-01	0.87 [0.51-1.47]	5.91E-01	0.72 [0.50-1.05]	9.07E-02
6	rs986475	31,556,709	G<A	4.55E-05	0.28	0.77 [0.65-0.92]	3.61E-03	0.65	0.61 [0.48-0.78]	4.94E-05	0.26	0.75 [0.60-0.93]	9.99E-03	0.81 [0.61-1.08]	1.54E-01	0.81 [0.47-1.39]	4.39E-01	0.57 [0.44-0.75]	3.51E-05
6	rs12199223	31,242,731	A<T	4.61E-05	0.95	1.33 [1.10-1.61]	2.79E-03	0.97	1.47 [1.13-1.92]	4.63E-03	0.92	1.33 [1.05-1.68]	1.95E-02	1.34 [0.99-1.82]	6.23E-02	1.51 [0.89-2.56]	1.29E-01	1.46 [1.07-1.99]	1.69E-02
6	rs113705304	32,590,453	G<C	4.84E-05	0.44	0.68 [0.56-0.83]	1.32E-04	0.17	0.78 [0.58-1.06]	1.14E-01	0.62	0.63 [0.50-0.79]	7.03E-05	0.85 [0.58-1.25]	4.18E-01	0.87 [0.52-1.48]	6.17E-01	0.74 [0.51-1.08]	1.15E-01
6	rs11752262	31,431,757	G<A	5.16E-05	0.88	0.73 [0.61-0.88]	9.00E-04	0.48	0.73 [0.55-0.95]	2.05E-02	0.67	0.70 [0.56-0.87]	1.60E-03	0.81 [0.58-1.13]	2.13E-01	0.63 [0.32-1.25]	1.88E-01	0.75 [0.56-1.00]	5.06E-02
6	rs58309024	33,046,438	G<A	5.24E-05	0.35	1.52 [1.17-1.97]	1.89E-03	0.42	2.29 [1.43-3.67]	5.78E-04	0.54	1.60 [1.19-2.14]	1.62E-03	1.21 [0.66-2.22]	5.39E-01	1.83 [0.78-4.28]	1.62E-01	2.53 [1.43-4.45]	1.35E-03
6	rs886390	30,334,994	A<C	5.41E-05	0.78	1.32 [1.14-1.53]	1.43E-04	0.88	1.19 [0.96-1.47]	1.14E-01	0.53	1.33 [1.12-1.58]	1.04E-03	1.30 [1.00-1.69]	5.29E-02	1.30 [0.91-1.86]	1.48E-01	1.13 [0.87-1.47]	3.69E-01
6	rs34723946	30,398,055	C<A	5.69E-05	0.56	1.35 [1.15-1.58]	1.85E-04	0.69	1.22 [0.96-1.56]	1.03E-01	0.23	1.38 [1.14-1.65]	7.22E-04	1.28 [0.95-1.72]	9.99E-02	1.47 [1.00-2.17]	4.98E-02	1.09 [0.79-1.48]	6.10E-01
6	rs12215313	32,441,276	T<C	5.81E-05	0.04	1.57 [1.41-1.76]	4.48E-15	0.81	1.25 [1.04-1.50]	1.88E-02	0.05	1.56 [1.37-1.78]	9.74E-12	1.62 [1.27-2.06]	1.00E-04	1.62 [1.18-2.23]	2.90E-03	1.09 [0.87-1.37]	4.52E-01
6	rs9275208	32,657,722	G<A	6.20E-05	0.14	0.73 [0.65-0.82]	3.88E-08	0.14	0.78 [0.66-0.93]	4.98E-03	0.09	0.69 [0.61-0.79]	4.02E-08	0.84 [0.67-1.05]	1.31E-01	0.62 [0.45-0.85]	3.43E-03	0.86 [0.70-1.05]	1.44E-01
6	rs9275210	32,657,817	C<T	6.20E-05	0.14	0.73 [0.65-0.82]	3.88E-08	0.14	0.78 [0.66-0.93]	4.98E-03	0.09	0.69 [0.61-0.79]	4.02E-08	0.84 [0.67-1.05]	1.31E-01	0.62 [0.45-0.85]	3.43E-03	0.86 [0.70-1.05]	1.44E-01
6	rs112723370	31,433,809	G<T	6.39E-05	0.85	0.73 [0.61-0.88]	9.57E-04	0.39	0.73 [0.55-0.96]	2.43E-02	0.87	0.69 [0.55-0.87]	1.25E-03	0.83 [0.59-1.16]	2.65E-01	0.69 [0.35-1.37]	2.88E-01	0.74 [0.55-0.99]	4.63E-02
6	rs6930444	30,763,632	G<A	6.55E-05	0.88	0.56 [0.42-0.75]	8.52E-05	0.89	0.72 [0.37-1.41]	3.34E-01	0.64	0.56 [0.41-0.76]	2.20E-04	0.59 [0.27-1.27]	1.79E-01	0.51 [0.10-2.49]	4.05E-01	0.77 [0.37-1.62]	4.97E-01
6	rs10081054	30,442,416	A<G	6.63E-05	0.43	1.16 [1.03-1.30]	1.06E-02	0.48	1.35 [1.13-1.60]	7.19E-04	0.64	1.13 [0.99-1.29]	6.84E-02	1.24 [1.00-1.54]	5.31E-02	1.26 [0.92-1.74]	1.52E-01	1.38 [1.13-1.70]	1.91E-03
6	rs9275101	32,649,355	G<A	6.67E-05	0.18	0.74 [0.66-0.83]	2.95E-07	0.24	0.79 [0.66-0.94]	9.38E-03	0.07	0.72 [0.63-0.82]	5.14E-07	0.84 [0.67-1.05]	1.18E-01	0.62 [0.45-0.85]	3.49E-03	0.88 [0.71-1.09]	2.36E-01

6	rs9275102	32,649,361	G<A	6.67E-05	0.18	0.74 [0.66-0.83]	2.95E-07	0.24	0.79 [0.66-0.94]	9.38E-03	0.07	0.72 [0.63-0.82]	5.14E-07	0.84 [0.67-1.05]	1.18E-01	0.62 [0.45-0.85]	3.49E-03	0.88 [0.71-1.09]	2.36E-01
6	rs17209950	32,446,588	A<G	6.84E-05	0.04	1.59 [1.42-1.78]	1.20E-15	0.93	1.25 [1.04-1.50]	1.84E-02	0.05	1.59 [1.40-1.81]	1.29E-12	1.57 [1.24-2.00]	2.12E-04	1.62 [1.18-2.23]	3.12E-03	1.10 [0.87-1.37]	4.28E-01
6	rs33987921	30,369,396	T<A	6.87E-05	0.56	1.31 [1.13-1.51]	2.24E-04	0.73	1.19 [0.97-1.47]	9.99E-02	0.22	1.33 [1.12-1.58]	1.11E-03	1.26 [0.97-1.63]	7.85E-02	1.43 [1.00-2.06]	5.15E-02	1.09 [0.84-1.41]	5.29E-01
6	rs1245219	30,347,910	A<G	6.93E-05	0.81	1.31 [1.13-1.51]	2.36E-04	0.79	1.20 [0.97-1.48]	9.66E-02	0.50	1.33 [1.12-1.57]	1.22E-03	1.27 [0.97-1.65]	7.82E-02	1.32 [0.92-1.89]	1.27E-01	1.14 [0.87-1.48]	3.50E-01
6	rs2395186	32,433,192	G<C	7.12E-05	0.04	1.58 [1.41-1.77]	1.75E-15	0.95	1.24 [1.03-1.49]	2.14E-02	0.05	1.58 [1.39-1.79]	2.79E-12	1.59 [1.25-2.02]	1.45E-04	1.60 [1.17-2.20]	3.74E-03	1.09 [0.87-1.37]	4.45E-01
6	rs2647045	32,668,100	A<G	7.59E-05	0.04	1.39 [1.24-1.57]	3.48E-08	0.99	1.47 [1.22-1.77]	5.68E-05	0.00	1.39 [1.22-1.59]	1.18E-06	1.40 [1.09-1.79]	9.09E-03	2.16 [1.56-2.99]	3.29E-06	1.21 [0.97-1.52]	9.81E-02
6	rs150110077	30,403,006	T<G	7.79E-05	0.53	1.35 [1.16-1.56]	9.39E-05	0.87	1.16 [0.92-1.45]	2.15E-01	0.32	1.36 [1.14-1.62]	7.43E-04	1.32 [1.00-1.74]	4.74E-02	1.34 [0.93-1.94]	1.21E-01	1.06 [0.79-1.41]	7.20E-01
6	rs6916921	31,520,426	T<C	7.86E-05	0.28	0.78 [0.66-0.93]	5.92E-03	0.67	0.61 [0.48-0.78]	5.31E-05	0.32	0.76 [0.61-0.95]	1.47E-02	0.82 [0.62-1.10]	1.80E-01	0.79 [0.46-1.36]	3.89E-01	0.58 [0.44-0.75]	4.58E-05
6	rs616187	32,574,305	A<G	7.90E-05	0.05	0.77 [0.69-0.86]	5.58E-06	0.15	0.61 [0.51-0.73]	9.21E-08	0.26	0.74 [0.65-0.84]	3.39E-06	0.89 [0.71-1.10]	2.79E-01	0.51 [0.36-0.73]	2.74E-04	0.65 [0.53-0.80]	4.66E-05
6	rs970270	30,347,306	A<G	8.03E-05	0.81	1.31 [1.13-1.50]	2.44E-04	0.85	1.19 [0.96-1.47]	1.08E-01	0.51	1.32 [1.11-1.56]	1.40E-03	1.28 [0.98-1.66]	6.85E-02	1.31 [0.92-1.88]	1.37E-01	1.13 [0.87-1.47]	3.69E-01
6	rs12214645	30,435,930	G<A	8.38E-05	0.47	1.34 [1.15-1.57]	2.45E-04	0.58	1.21 [0.96-1.54]	1.13E-01	0.19	1.38 [1.15-1.67]	6.66E-04	1.25 [0.93-1.69]	1.41E-01	1.49 [1.01-2.19]	4.42E-02	1.07 [0.79-1.45]	6.69E-01
6	rs56276901	33,049,003	G<A	9.16E-05	0.63	1.47 [1.14-1.90]	3.17E-03	0.31	1.81 [1.18-2.79]	7.02E-03	0.86	1.57 [1.18-2.09]	1.96E-03	1.12 [0.63-2.00]	6.94E-01	1.71 [0.79-3.72]	1.74E-01	1.86 [1.11-3.13]	1.96E-02
6	rs35102994	30,359,293	G<A	9.25E-05	0.78	1.30 [1.13-1.50]	2.68E-04	0.85	1.19 [0.96-1.47]	1.14E-01	0.46	1.32 [1.11-1.56]	1.51E-03	1.28 [0.98-1.66]	7.09E-02	1.32 [0.92-1.90]	1.26E-01	1.12 [0.86-1.46]	4.02E-01
6	rs9394007	30,452,962	G<T	9.37E-05	0.38	1.15 [1.03-1.29]	1.25E-02	0.43	1.35 [1.14-1.61]	6.49E-04	0.64	1.12 [0.98-1.28]	8.34E-02	1.25 [1.00-1.55]	4.87E-02	1.27 [0.92-1.74]	1.45E-01	1.39 [1.13-1.70]	1.84E-03
6	rs9380180	30,452,963	A<C	9.37E-05	0.38	1.15 [1.03-1.29]	1.25E-02	0.43	1.35 [1.14-1.61]	6.49E-04	0.64	1.12 [0.98-1.28]	8.34E-02	1.25 [1.00-1.55]	4.87E-02	1.27 [0.92-1.74]	1.45E-01	1.39 [1.13-1.70]	1.84E-03
6	rs374842482	31,422,323	A<G	9.71E-05	0.35	2.16 [1.54-3.02]	7.23E-06	0.74	1.37 [0.83-2.24]	2.16E-01	0.32	2.21 [1.54-3.17]	1.81E-05	1.87 [0.76-4.60]	1.71E-01	1.07 [0.54-2.14]	8.39E-01	1.77 [0.87-3.61]	1.16E-01

Supplementary Table S2. Discovery and replication results of the *IL12B* rs755374 association with large vessel vasculitis.

Trait	Study	P-value	OR [95% CI]	Q
GCA	Spain	**7.29E-03**	1.20 [1.05-1.38]	
	Italy	2.57E-01	1.14 [0.91-1.43]	
	Combined	**3.92E-03**	1.19 [1.06-1.33]	0.69
TAK	North America	5.20E-02	1.36 [1.00-1.86]	
	Turkey	**2.57E-05**	1.56 [1.27-1.93]	
	Combined	**4.71E-06**	1.50 [1.26-1.78]	0.47
TAK/GCA	Meta-analysis (Discovery)	**7.54E-07**	1.28 [1.16-1.41]	0.14
GCA	Replication*	**4.69E-02**	1.13 [1.01-1.27]	0.54
	Discovery + Replication*	**5.52E-04**	1.16 [1.07-1.26]	0.75
TAK/GCA	Overall Meta-analysis	**3.41E-07**	1.22 [1.13-1.31]	0.19

*N = 650 GCA cases and 12,491 controls (from UK, USA/Canada, Germany and Norway)
